# Developmental Gene Discovery in a Hemimetabolous Insect: *De Novo* Assembly and Annotation of a Transcriptome for the Cricket *Gryllus bimaculatus*

**DOI:** 10.1371/journal.pone.0061479

**Published:** 2013-05-06

**Authors:** Victor Zeng, Ben Ewen-Campen, Hadley W. Horch, Siegfried Roth, Taro Mito, Cassandra G. Extavour

**Affiliations:** 1 Department of Organismic and Evolutionary Biology, Harvard University, Cambridge, Massachusetts, United States of America; 2 Departments of Biology and Neuroscience, Bowdoin College, Brunswick, Maine, United States of America; 3 Institute for Developmental Biology, University of Cologne, Cologne Biocenter, Cologne, Germany; 4 Department of Life Systems, Institute of Technology and Science, The University of Tokushima Graduate School, Tokushima City, Japan; University of Otago, New Zealand

## Abstract

Most genomic resources available for insects represent the Holometabola, which are insects that undergo complete metamorphosis like beetles and flies. In contrast, the Hemimetabola (direct developing insects), representing the basal branches of the insect tree, have very few genomic resources. We have therefore created a large and publicly available transcriptome for the hemimetabolous insect *Gryllus bimaculatus* (cricket), a well-developed laboratory model organism whose potential for functional genetic experiments is currently limited by the absence of genomic resources. cDNA was prepared using mRNA obtained from adult ovaries containing all stages of oogenesis, and from embryo samples on each day of embryogenesis. Using 454 Titanium pyrosequencing, we sequenced over four million raw reads, and assembled them into 21,512 isotigs (predicted transcripts) and 120,805 singletons with an average coverage per base pair of 51.3. We annotated the transcriptome manually for over 400 conserved genes involved in embryonic patterning, gametogenesis, and signaling pathways. BLAST comparison of the transcriptome against the NCBI non-redundant protein database (**nr**) identified significant similarity to **nr** sequences for 55.5% of transcriptome sequences, and suggested that the transcriptome may contain 19,874 unique transcripts. For predicted transcripts without significant similarity to known sequences, we assessed their similarity to other orthopteran sequences, and determined that these transcripts contain recognizable protein domains, largely of unknown function. We created a searchable, web-based database to allow public access to all raw, assembled and annotated data. This database is to our knowledge the largest *de novo* assembled and annotated transcriptome resource available for any hemimetabolous insect. We therefore anticipate that these data will contribute significantly to more effective and higher-throughput deployment of molecular analysis tools in *Gryllus*.

## Introduction

The vast majority of existing insect genomic resources are for the Holometabola or “higher insects,” which undergo true metamorphosis. These include disease vectors such as the mosquito *Anopheles gambiae* [1], agricultural pests such as the flour beetle *Tribolium castaneum* [2], and the powerful genetic model organism *Drosophila melanogaster* [3], [4]. However, there are very few complete genome sequences available for the Hemimetabola or “lower insects”, which do not undergo true metamorphosis and branch basally to the Holometabola. Only three of the over 146,000 estimated species of hemimetabolous insects [5] have available genome sequences: the aphid *Acyrthosiphon pisum* [6], the kissing bug *Rhodnius prolixus* [7], [8], and the human body louse *Pediculus humanus* [9]. Moreover, sequence divergence is so great among insects [10] that a specific genome cannot be used as a reference sequence for other insects even within the same order; see for example [11].

Among the Hemimetabola, the basally branching orthopteroid orders of insects are of particular interest to many fields of biology. Orthopterans have served as classical model organisms for neurobiology for several decades [12]. Multiple cricket species have been used for important studies of ecologically relevant polyphenisms (reviewed in [13]), the evolution of endocrine functions and photobiology [14], [15], [16], [17], speciation [18], [19], [20], [21], [22] and the evolution of behavior [23], [24], [25]. Crickets and locusts have also been important for addressing outstanding questions in evolutionary developmental biology, such as the evolution of molecular mechanisms for regeneration, segmentation, and axial patterning [26], [27], [28], [29], [30], [31], [32], [33]. However, *de novo* genome assembly for organisms with extremely large genome sizes is costly and challenging [34], [35], [36]. Grasshopper genomes can be over twice as large as the human genome [37], and even the genome of the laboratory model cricket *Gryllus bimaculatus* is estimated at 1.7 Gbp (C. G. Extavour and R. Gregory, unpublished). If orthopteran genome projects are eventually undertaken, their annotation success will be significantly enhanced by the availability of large transcriptomes, but these are also few in number.

To date, only three Sanger-based EST projects and one large *de novo* assembled transcriptome generated with next-generation sequencing have been reported for orthopterans (Table 1). These projects have focused on specific post-embryonic developmental stages of pest locusts (*L. migratoria*, *S. gregaria*) and on the CNS of a cricket (*L. kohalensis*). Although most functional genetic studies on orthopterans focus on embryonic development (see for example [28], [29], [38], [39]) and neurophysiological studies are increasingly examining the embryonic origins of neural structures and functions (see for example [e.g. 16], [40], [41], [42], [43]), a transcriptome enriched for embryonic developmental transcripts is lacking. Here we present such a transcriptome for the model laboratory cricket, *G. bimaculatus*.

**Table 1:** Large-scale Orthopteran transcriptome resources to date.

	*Locusta migratoria* 1	*Laupala kohalensis* 2	*Schistocerca gregaria* 3	*Locusta migratoria* 4	*Gryllus bimaculatus* 5
**Orthopteran Suborder**	Caelifera	Ensifera	Caelifera	Caelifera	Ensifera
**Superfamily**	Acridoidea	Grylloidea	Acridoidea	Acridoidea	Grylloidea
**Family**	Acrididae	Gryllidae	Acrididae	Acrididae	Gryllidae
**Sequencing Platform**	Sanger	Sanger	Sanger	Illumina	454 Titanium
**Tissue Source(s)**	L5^6^	L5–L8 CNS	L3–L5 & adult CNS	Mainly L4	Ovaries & embryos
**Normalized Library**	No	Yes	Yes	No	No
**# Raw Reads**	76,012	14,502	nd	447,718,464	4,248,346
**# Reads Used in Assembly**	45,449	14,377	34,672	nd	4,216,721
**# bp Used in Assembly**	21,760,812	10,121,408	nd	nd	1,449,059,795
**% Raw Reads Assembled**	59.79%	99.14%	nd	nd	99.26%
**# Contigs or Clusters**	4,550	2,575	4,785	72,977	43,321
**N50** 7 **or Mean Contig Length (bp)**	471	935	750	2,275	2,133
**# Singletons or # Single ESTs** 8	7,611	6,032	7,924	nd	120,805
**% Singletons (of assembled reads)**	16.75%	41.96%	22.85%	nd	2.86%
**# Total Assembly Products**	12,161	8,607	12,709	72,977	142,317
**# Unigenes or # Unique BLAST** 9 **Hits to nr**	**12,616**	**8,607**	**12,709**	**11,490**	**19,874**

1 Data from [73], [74].

2 Data from [75].

3 Data from [76].

4 Data from [72].

5 Data from this report.

6 L = larval stage. nd = data not reported in the relevant publication [72], [73], [74], [76].

7 “N50” refers to isotig N50 from the *G. bimaculatus de novo* transcriptome assembly; mean contig length is shown for all other orthopteran transcriptome resources in this table.

8 # singletons are shown for the *G. bimaculatus de novo* transcriptome assembly; # single ESTs (not incorporated into contigs) are shown for all other orthopteran transcriptome resources in this table.

9 # unique BLAST hits against **nr** are shown for the *G. bimaculatus de novo* transcriptome assembly; # unigenes are shown for all other orthopteran transcriptome resources in this table.

**Figure 1:**
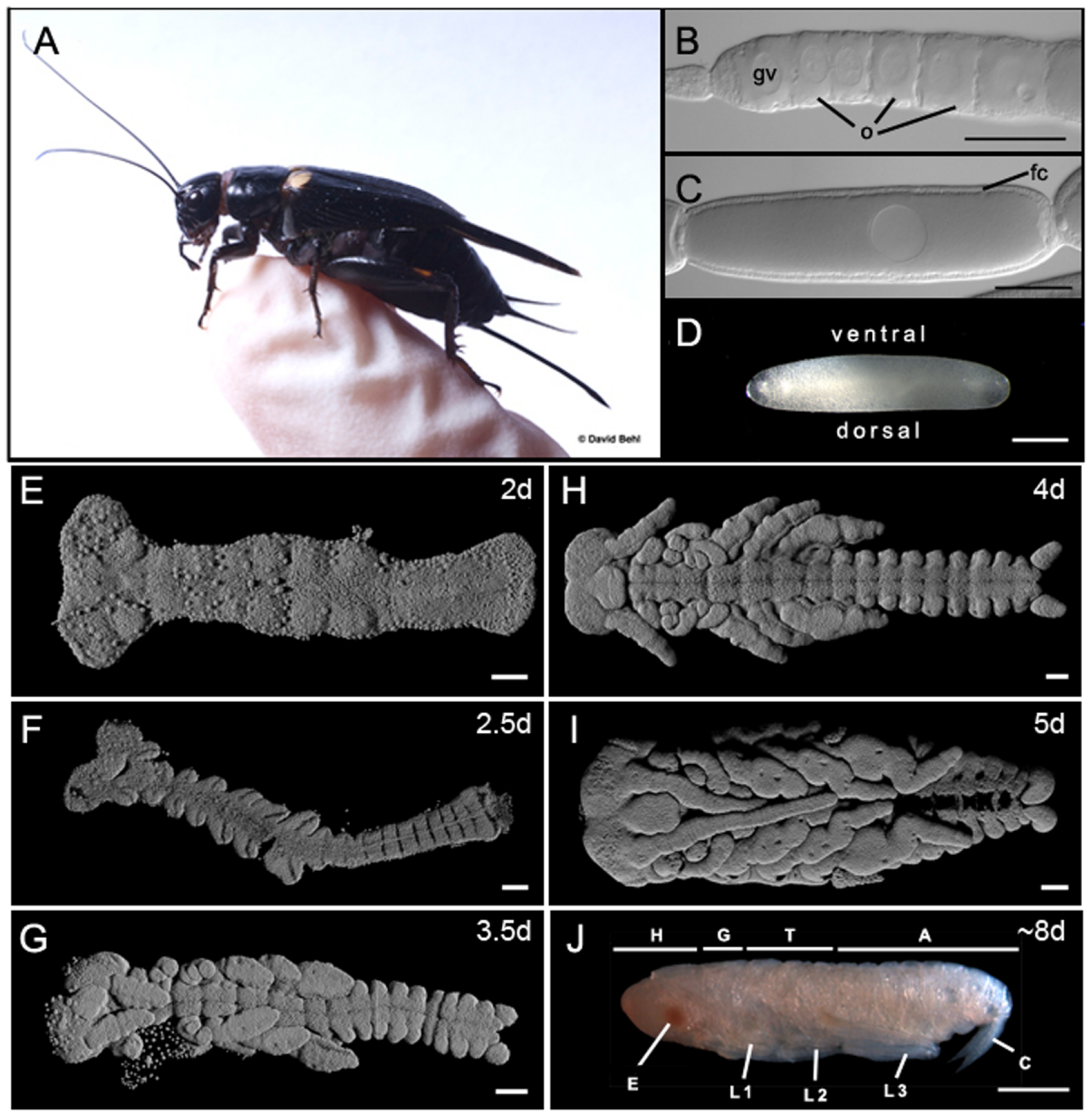
Oogenesis and embryogenesis in the cricket model organism *Gryllus bimaculatus*. (A) Adult female cricket perched on a gloved human finger for perspective. (B) Anterior tip of a single ovariole from an adult female ovary, showing oocytes (o) at early previtellogenic stages of oogenesis. A single large germinal vesicle (gv) is distinguishable in each oocyte. Unlike meroistic (containing nurse cells) *Drosophila* ovaries, *G. bimaculatus* ovaries are panoistic and lack nurse cells [100]. (C) A single late stage oocyte with a single layer of columnar follicle cells (fc). (D–J) Chronological stages of *G. bimaculatus* embryogenesis showing the range of embryonic stages represented in the transcriptome presented here. (D) A fertilized egg just after laying. The egg nucleus is distinguishable as a dense patch in the dorsal yolk (arrowhead). Ages are shown as days (d) after egg-laying at 29°C. (E–I) are 3D reconstructions of confocal optical sections of Hoechst 3342-stained embryos dissected free from the egg; (J) is a micrograph of a live embryo dissected free from the chorion. Abbreviations: A = abdomen; C = cerci; E = eye; H = head; G = gnathal segments; L1 = first thoracic leg; L2 = second thoracic leg; L3 = third thoracic leg; T = thorax. Scale bar is 100 µm in (B, C, E–I) and 500 µm in (D, J). Anterior is to the left in all panels. Photo in (A) courtesy of David Behl; photos in (D) and (J) from [101].

*G. bimaculatus* is a highly tractable orthopteran model for functional genetic studies in the laboratory (Fig. 1). Gene knockdown can be achieved by RNA interference during embryonic, post-embryonic and regenerative development [32], [43], [44]. *G. bimaculatus* is also the only orthopteran for which stable germ line transgenesis has been established [39]. Moreover, protocols for targeted genome editing using zinc finger nucleases or TALE nucleases have recently been developed [45]. However, all *G. bimaculatus* genes studied to date have been obtained by degenerate PCR (for example [28], [46]) or from limited Sanger-based EST libraries or RNA-Seq data that are not available in an annotated database (for example [26], [47]).

In this report we present a *de novo* assembled and annotated transcriptome for *G. bimaculatus* oogenesis and embryonic development. We show that this transcriptome contains more putative unique gene transcripts than previous orthopteran transcriptomes, and adds sequence data to known GenBank accessions for *G. bimaculatus*. We manually annotate over 400 developmental genes, and develop an automated annotation method for the entire transcriptome based on similarity to *Drosophila* sequences. For predicted transcripts that lack significant similarity to GenBank accessions, we examine specifically those that are more similar to known orthopteran sequences, and find that the most represented predicted protein domains of such “orthopteroid” transcripts are domains of unknown function (DUFs). In contrast, the most represented predicted protein domains of transcripts of the transcriptome overall are zinc finger domains. Finally, we created a publicly accessible repository and database for the transcriptome, which is searchable by BLAST, pre-computed BLAST hits, or putative orthology assignments (gene names) derived from both manual and automated annotation.

## Materials and Methods

### Animal culture and collection of tissues for cDNA synthesis

*G. bimaculatus* cultures were maintained as previously described [28], at 28–29°C on a diet of oatmeal, wheat germ, soya protein, corn meal, sugar, yeast, salt, corn oil and Purina Cat Chow. This non-isogenic culture derives from a population of *G. bimaculatus* obtained from Livefoods Direct (Sheffield, UK), and was maintained as an inbred, self-sustaining culture for four years (or approximately 26 generations) prior to tissue collection. We do not have estimates of genetic polymorphism for this population, so that accurate interpretation of putative SNP data is not possible in the present analysis. Separate egg collections (total mass 781 mg) of 50–100 embryos on each of the first eight days of embryogenesis (approximately 66.7% of development at 28°C) (Figure 1D–J) were washed in distilled water, shock frozen in liquid nitrogen and stored at −80°C. Embryos were collected from cages containing 25–50 females per cage. Ovaries from one adult female (Fig. 1B, C) were dissected from the body cavity, rinsed in 1× PBS, and homogenized in TRIzol (Invitrogen, NY, USA).

### cDNA Synthesis

Total RNA was isolated separately from embryos at each day of embryonic development and from ovaries, using TRIzol (Invitrogen, NY, USA) and following manufacturer's instructions. RNA isolation was performed separately from embryonic and ovarian tissues, so that tissue lysis, which can affect the efficiency of subsequent RNA isolation, would be as homogeneous as possible within a sample. A pilot study was first conducted to determine library quality by sequencing ovarian and embryonic cDNA separately. For this pilot sequencing run, cDNA was synthesized using the SMART cDNA synthesis kit (Clontech, CA, USA) and normalized using the Evrogen Trimmer Direct kit (Evrogen, Moscow, Russia) following previously described methods [11]. Results from both libraries were comparable in read length and sequence quality, and all further experiments were carried out with pooled RNA libraries as described below. Raw reads from the pilot studies were incorporated into the final assembly as previously described [11].

To create a pooled cDNA library for large-scale sequencing, 1.5 µg of each of the mixed-stage embryonic RNA pool and ovarian RNA was used as a template for first strand cDNA synthesis. cDNA was synthesized as previously described [11]. Primary amplification proceeded with 10 PCR cycles monitored in real-time via qPCR [22], and secondary amplification began to plateau after 9 cycles. 16 parallel reactions of 0.73 µg each were co-purified into elution buffer using QIAquick PCR purification columns (Qiagen Inc., CA, USA). These 16 parallel reactions were identical, and were performed in individual tubes for the sole reason that a single PCR reaction sufficient to generate the 2 µg of cDNA required for sequencing would have had to be performed in a volume too large to undergo efficient cycling in our PCR machine (Bio-Rad Tetrad 2). We therefore calculated the predicted yield from the largest single PCR reaction that we could perform in our machine, and scaled up the number of reactions in parallel to achieve the required 2 µg total yield.

### 454 Titanium Pyrosequencing

The samples were nebulized, adaptor-ligated, and pyrosequenced using the 454 GS-FLX platform on pilot embryonic and ovarian cDNA separately, or the 454 GS-FLX Titanium platform for pooled ovarian/embryonic cDNA samples by the Institute for Genome Science and Policy DNA Sequencing Facility (Duke University). All of the raw reads generated in this study have been submitted to the NCBI Short Read Archive (Study Accession Numbers SRX023831, SRX023830, and SRX023832).

### Sequence Assembly

Sequences were trimmed and assembled with Newbler v2.5, which was shown to outperform other assemblers for *de novo* assembly of 454 pyrosequencing reads [48]. Assembly parameters are described in [49], with the exception of the file used for the –vt flag (“Gb Adaptors”), which is available at http://www.extavourlab.com/protocols/index.html. Assembly results are available at http://www.extavourlab.com/resources/index.html and at http://asgard.rc.fas.harvard.edu/download.html).

### Sequence Annotation

A nucleotide BLAST database was created using the isotigs and singletons produced by the Newbler assembly. To increase efficiency of BLAST comparison to this database, we first removed redundant isotigs and singletons created due to a combination of putative SNPs, sequencing errors, and low quality reads. Note that these data could in principle yield SNP data, but as we did not use an isogenic *G. bimaculatus* culture, nor do we have estimates of polymorphism for the culture, an accurate SNP analysis is not performed in the present study. Each assembly product was compared with the BLAST database using the BLASTN algorithm. Individual isotigs and singletons with BLAST hits (>95% identity based on bit score and sequence length) to longer sequences in the assembly, resulting in a high scoring segment pair (HSP) that spans the full length of the sequence, were removed. To identify the number of unique BLAST hits we followed the method described in [49].

To identify members of signaling pathways as described by the KEGG database [50], we manually annotated the *G. bimaculatus* transcriptome as described in [49]. Briefly, BLAST was used to compare the sequences of *D. melanogaster* pathway members with the *G. bimaculatus* transcriptome assembly and the top hit was selected as a putative ortholog with an E-value cutoff of e-10.

To determine whether the *de novo* assembly contained members of previously known *G. bimaculatus* GenBank accessions, we used tBLASTn (for 80 protein coding genes) or BLASTn (for 3 ribosomal RNA genes) to query the *G. bimaculatus* transcriptome assembly.

For automatic annotation of all transcriptome sequences, we designed a custom script called “Gene Predictor” (genePrediction.pl, available at http://www.extavourlab.com/protocols/index.html). This script assigns putative gene orthology based on comparisons with the *D. melanogaster* proteome, downloaded as described in Table S1. A protein BLAST database was created using the *D. melanogaster* proteome. A nucleotide BLAST database was created using the non-redundant assembly products (isotigs and singletons) of the *G. bimaculatus de novo* transcriptome assembly. The top 50 BLAST hits for each sequence of the *D. melanogaster* proteome compared with the *G. bimaculatus* transcriptome were obtained using the TBLASTN algorithm and stored in a MySQL database. Reciprocally, the top BLAST hit for each sequence of the *G. bimaculatus* transcriptome against the *D. melanogaster* proteome was obtained using the BLASTX algorithm and stored within a separate MySQL database. A custom script then iterates through each of the entries of the *D. melanogaster* proteome vs. the *G. bimaculatus* transcriptome MySQL database indices based on query identity and e-value. The same script also checks the *G. bimaculatus* transcriptome sequence identity against the *D. melanogaster* proteome MySQL database to confirm if the reciprocal top BLAST hit is the same as the *D. melanogaster* query. After confirmation of the reciprocal BLAST identity, the script verifies whether any *G. bimaculatus* transcriptome sequences have already been assigned to the same *D. melanogaster* protein. If the existing sequence does not overlap with the confirmed sequence for more than 14 amino acids based on their HSP against the *D. melanogaster* protein, both sequences are recorded as orthologs. Otherwise, the confirmed sequence is further processed to determine whether it is a putative isoform or paralog of the existing sequence. If the confirmed sequence is a singleton or in the same isogroup as the existing sequence based on Newbler prediction, it is designated as an alternate isoform; otherwise, the sequence is annotated as a putative paralog.

A list of all curated *D. melanogaster* transcription factors was downloaded on March 26^th^ 2011 from http://flytf.org. Each *D. melanogaster* transcription factor was examined to determine whether it was predicted to have an ortholog in the *G. bimaculatus* transcriptome using the Gene Predictor script described above. Custom scripts to generate tables based on the ASGARD schema (“ASGARD_NEW_DB.pl”) [51], upload assembled transcriptome sequences into ASGARD tables (“ASGARD_UPLOAD.pl”), upload BLAST results of the D. melanogaster proteome against the assembled transcriptome (“up_DMP.pl”), upload the BLAST results of the assembled transcriptome against the D. melanogaster proteome (“up_vDMP.pl”), and determine the best reciprocal BLAST result for each assembly products (“gene_prediction.pl”) are available at http://www.extavourlab.com/protocols/bio_tools/ASGARD_uploadGene_Predictor.zip).

### Determination of sequencing depth and transcript completion

Ortholog hit ratio calculations and subassembly experiments were performed as described in [49]. Briefly, ortholog hit ratios were calculated using a custom script (“OrthologHitRatio.pl” available at http://www.extavourlab.com/protocols/bio_tools/Perl_Transcriptome_Analysis_Scripts.zip) that compares the length of each assembly product with the full length of its putative orthologous mRNA in *D. melanogaster*, based on the reciprocal best BLAST hit criteria described above. Subassemblies were performed by assembling progressively larger random subsets of all trimmed reads, using the same assembly parameters as those used for the complete assembly.

### Protein Domain Analysis

23 proteomes based on completely sequenced genomes and two EST libraries were downloaded as described in Table S1. A protein BLAST database was created from each proteome. All *G. bimaculatus* assembly products were compared with each database using the BLASTX algorithm with an E-value cutoff of 1e-5. The resulting reports were parsed using the Uniqueblast.pl script as previously described [49] (available at http://www.extavourlab.com/protocols/index.html).

A local installation of EST Scan [52] (ESTSCAN 3.03) was downloaded on April 11^th^ 2011 as a Linux rpm package from http://estscan.sourceforge.net/. All assembly products were screened using ESTSCAN with default parameters, except for the “-l” flag that was used with a value of 20 to restrict the minimum result size to 20 amino acids. The “-t” flag was also used to allow ESTSCAN to produce the predicted protein sequence of each assembly product.

A local installation of InterPro Scan [53], [54] (IPRSCAN 4.7) was downloaded on April 15^th^ 2011 from ftp://ftp.hgc.jp/pub/mirror/ebi/software/iprscan/index.html. The “-cli” flag was used to turn on pipeline mode and suppress html outputs. All assembly products were screened using IPRSCAN against existing protein feature databases [55], [56], [57], [58], [59], [60], [61], [62], [63], [64], [65], [66], and the results were stored in xml format for further analysis.

Welch's t-test (appropriate in this case for use with samples with unequal variance [67]) was used for statistical comparisons of lengths of sequences and predicted protein coding regions in various annotation categories.

## Results and Discussion

### Collection and preparation of material

We aimed to create a transcriptome containing genes deployed during oogenesis, when maternally deposited factors required for embryogenesis may be synthesized, and during all stages of embryogenesis. We therefore collected ovaries (Figure 1B, C) and embryos from early to late stages of embryogenesis (Figure 1D–J) for mRNA extraction. We pooled these mRNA samples and prepared non-normalized cDNA libraries for 454 Titanium pyrosequencing. We chose to omit normalization in preparing these libraries as our previous studies [11] suggest that at this scale of sequencing, normalization does not significantly aid in gene discovery.

### Sequencing and basic transcriptome assembly

We used Newbler v2.5 (Roche) for the *de novo* assembly of 4,248,348 raw reads (1,483,726,666 bp) obtained by 454 pyrosequencing (Table 1). Using default Newbler assembly parameters, raw reads were screened and trimmed of both 5′ and 3′ adaptors (see Methods), and low quality reads were removed. (Newbler's quality scores are defined as “Phred-like” or “Phred equivalent” [68]. The Phred quality score is a widely used base quality parameter defined by determining qualities of the data used to generate each base call [69], [70]. We used a Newbler quality score cutoff of >20; a Phred score of 20 would indicate a base call accuracy of ≥99%.). 99.26% of all reads passed this quality control process (4,216,721 reads = 1,449,059,795 bp) (Figure S1A, Table 1), and were subsequently used in the sequence alignment process. 88.78% of these reads (3,743,561) were fully assembled, meaning that the entire read sequence was used in a contig. 6.69% (282,259) were partially assembled, meaning that the entire read was not used in a contig (Figure S1B, C). Of the 190,901 good quality reads (4.53%) that were not aligned, 13,416 (0.32%) were too short (<40 bp) to be included in the assembly, 1,989 (0.05%) were predicted to be from a repeat region (meaning that >70% of the read's seeds match at least 70 other reads, or determined to partially overlap a contig; note that portions of reads in this category that overlap unique contigs are still included in the assembly results), 54,691 (1.30%) were considered outliers (e.g. chimeric reads or results of sequencing errors), and 120,805 (2.86%) were preserved as singletons.

Newbler assembly products fall into one of four categories: (1) *contigs* are groups of assembled reads with significant overlapping regions (we used the Newbler default minimum overlap of 40 bp), which may represent exons; (2) *isotigs* are continuous paths through a given set of contigs, and represent putative transcripts, including possible splice variants of a given transcription unit; (3) *isogroups* are groups of isotigs that were assembled from the same contig set, and are the closest to gene predictions as it is possible for a *de novo* assembly to achieve; and (4) *singletons*, which are single good quality reads that lack significant overlap with any other read, and therefore are not incorporated into any contig. We use these terms henceforth to refer to the *G. bimaculatus* assembly products. It is important to note that determination of whether contigs represent true exons, or isotigs true transcripts, would require further validation by sequencing full-length cDNAs and comparison with a fully sequenced genome. For this reason we refer to the *G. bimaculatus* transcriptome *de novo* assembly products as “contigs” and “isotigs” or “predicted transcripts” or “putative transcripts” throughout, rather than as “exons” or “transcripts” respectively.

Upon assembly we obtained 43,321 unique contigs using the aligned reads (Table 1). Newbler then further assembled these contigs into 21,512 isotigs that belonged to 16,456 isogroups (Table 2). 13,157 (79.95%) of the isogroups (putative genes) consist of only a single isotig, and on average there are 1.2 isotigs per isogroup (Table 2). 12,701 (62.78%) isotigs consist of a single contig, and on average there are 1.7 contigs per isotig. The isotig N50 is 2,133 bp (Table 1), meaning that the majority of predicted transcripts are over 2 kb in length. FASTA files of all assembly products are available for download from our interactive database (described below).

**Table 2:** Assembly statistics and BLAST results against nr for the *G. bimaculatus de novo* transcriptome assembly.

Parameter^1^	Value
# bp Raw reads	1,483,726,666
Maximum raw read length	803
Minimum raw read length	13
Median raw read length	364
Maximum assembled read length	771
Minimum assembled read length	20
Median assembled read length	358
# Isogroups^2^ (“genes”)	16,456
Mean # isotigs per isogroup	1.2
# Isotigs	21,512
Maximum isotig length	10,865
Minimum isotig length	57
Median isotig length	1,054.5
# Isotigs with BLAST hit against **nr** 3, E-value cutoff e-10 (% of all isotigs)	11,135 (51.8%)
# Isotigs with BLAST hit against **nr**, E-value cutoff e-5 (% of all isotigs)	11,943 (55.5%)
Mean # contigs per isotig	1.7
# Singletons	120,805
Maximum singleton length	620
Minimum singleton length	50
Median singleton length	250.5
# Singletons with BLAST hit against **nr**, E-value cutoff e-10 (% of all singletons)	7,914 (6.6%)
# Singletons with BLAST hit against **nr**, E-value cutoff e-5 (% of all singletons)	10,815 (9.0%)
# Non-redundant assembly products (NRAP)	142,317
# NRAP with BLAST hit against **nr**, E-value cutoff e-10 (% of all NRAP)	19,049 (13.4%)
# NRAP with BLAST hit against **nr**, E-value cutoff e-5 (% of all NRAP)	22,758 (16.0%)
Total # BLAST hits^4^ (**nr**)	22,758
Average coverage/bp	51.3

1 Values for number of raw reads, number and % of raw reads assembled (passed quality filters described in main text), number of contigs, isotig N50, % of singletons, total number of assembly products, and number of unique BLAST hits against **nr**, are shown in Table 1.

2 Because isogroups are collections of isotigs that are hypothesized to originate from the same gene, they do not comprise a single sequence and so cannot be mapped to **nr** using BLAST.

3 **nr** = NCBI non-redundant database.

4 For BLAST against **nr** the E-value cutoff was 1e-5. For breakdown of BLAST hits among different classes of assembly sequences, see Table 3.

### Assessment of transcript coverage and depth

The average coverage across the assembly is 51.3 reads per base pair; in other words, each base pair of the assembly was sequenced on average over 50 times. This coverage is high compared to other *de novo* transcriptome assemblies [11], [49], [71], which we attribute largely to the high number of reads used to create the *G. bimaculatus* transcriptome. We note, however, that the *G. bimaculatus* transcriptome coverage we obtained is more than twice as high as that of the recently *de novo* assembled transcriptome for the crustacean *Parhyale hawaiensis* (25.4 reads/bp), even though the *G. bimaculatus* transcriptome contained only 1.3 fold more base pairs in raw reads than that of *P. hawaiensis*, which was also generated from embryonic and ovarian cDNA, and was assembled and annotated identically to the *G. bimaculatus* transcriptome described in this report [49].

**Figure 2:**
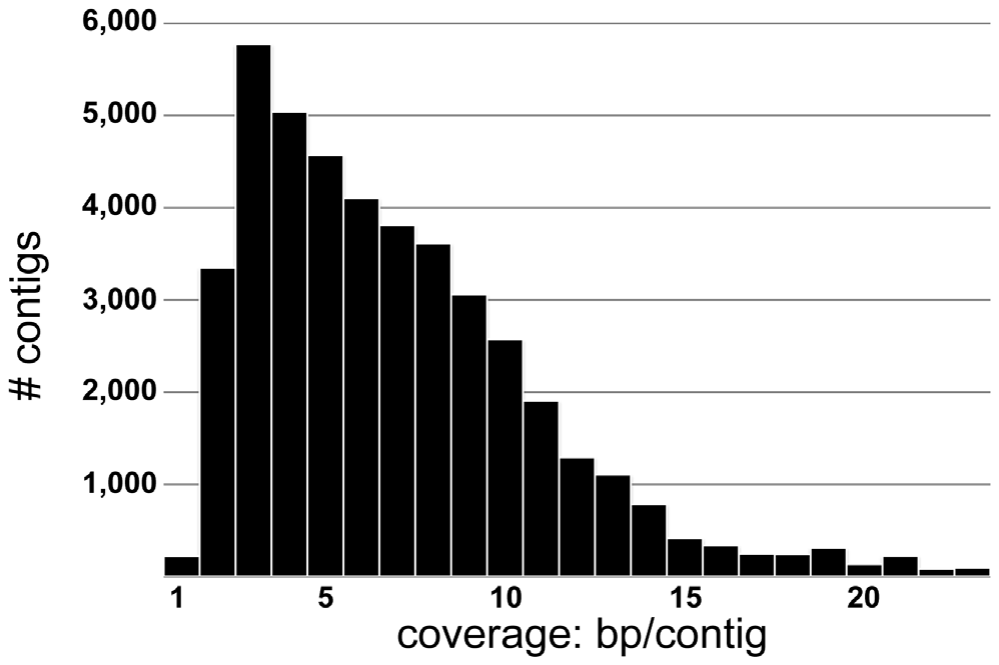
Distribution of average coverage (bp/contig) within contigs produced by *de novo* assembly of the *G. bimaculatus* transcriptome. The coverage within contigs is calculated by dividing the total number of base pairs contained in the reads used to construct a contig by the length of that contig.

An additional measure of coverage is the average contig read depth (total number of base pairs from all reads aligned to generate a given contig, divided by contig length). This value is 391 bp/contig, with a median value of 16.7 bp/contig. We note that the predicted transcript coverage (number of base pairs of raw reads comprising each contig) is highly variable, suggesting that some genes are represented by many more raw reads than others (Figure 2). 19,093 (43.97%) contigs had a coverage ≤10 bp/contig, and 538 contigs (1.24%) had a coverage ≥10,000 bp/contig.

**Figure 3:**
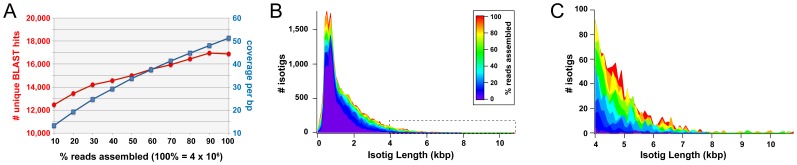
Assessment of gene discovery and read length capacity of the *G. bimaculatus de novo* assembled transcriptome. (A) Randomly selected subsets of the trimmed reads were assembled using Newbler v2.5 in 10% increments, up to and including 100% of trimmed reads. For each subassembly, the number of unique BLAST hits against the NCBI non-redundant database (**nr**) with an E-value cutoff of 1e-10 (red; left axis) and the average coverage per base pair (blue; right axis) was calculated (see text for details). The number of unique BLAST hits did not increase after at least 90% of reads (3,795,085 reads) were assembled, while the coverage per base pair continued to increase as reads were added to the assembly. (B) Isotig length distribution for each subassembly created as described in (A). (C) Isotig length distribution of each subassembly for isotigs ≥4 kb. High numbers (≥50) of isotigs over 4 kb in length are achieved only when ≥40% of reads (1,686,646 reads) are assembled.

We wished to determine whether similar coverage levels and predicted transcript lengths could have been obtained with fewer reads, and how well our transcriptome had identified all putative transcripts present in our samples. To do this, we created subassemblies using randomly chosen subsets of reads, starting with 10% of reads and adding increments of 10% up to the full complement of trimmed reads. For each subset of reads, we performed an independent assembly with Newbler v2.5. For each of these nine subassemblies, we then assessed both read length distribution and the number of unique BLAST hits against the NCBI non-redundant protein database (**nr**) with an E-value cutoff of 1e-10. The mean coverage per bp was strongly positively correlated (R^2^ = 0.96, linear regression) with the number of reads used for the assembly (Figure 3A, blue line). We also found that as the number of reads used in the subassembly increased, the proportion of reads left as singletons decreased from 11.25% for the 10% subassembly, to 2.86% in the full assembly. This is likely because contigs and isotigs increased in length as reads were added (Figure 3B), as we observed an increase in isotig N50 from 1,290 bp with 10% of reads to 2,133 bp with all reads. The distribution of isotig lengths in each subassembly (Figure 3B) indicates the maximum length of assembled isotigs given a certain number of reads. A small proportion of isotigs exceeding 4 kb can be obtained with only 10% of all reads, but by assembling all reads it was possible to obtain predicted transcripts exceeding 10 kb (Figure 3C).

The number of unique BLAST hits against **nr** obtained from all isotigs also increased with the number of reads (Figure 3A, red line), but at a slower rate than that of mean coverage per bp (Figure 3A, blue line). Slightly fewer unique BLAST hits were obtained from isotigs generated with 100% of reads compared to 90%, which may mean that previously unconnected contigs were increasingly incorporated into isotigs as they increased in length and acquired overlapping regions.

**Figure 4:**
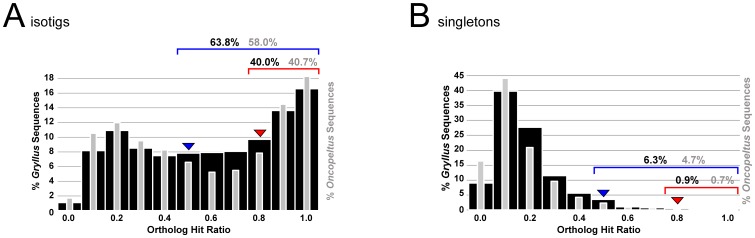
Ortholog hit ratio analysis of the *G. bimaculatus de novo* assembled transcriptome. The ortholog hit ratio is a comparison of the length of an assembled sequence to the total length of the full length transcript of its putative ortholog [71]. Values close to one suggest that a transcript predicted by the *de novo* assembly is close to full length. Ortholog hit ratios for the *G. bimaculatus* transcriptome sequences are compared to those for the previously reported *de novo* assembled transcriptome of another insect, the milkweed bug *Oncopeltus fasciatus* [11]. (A) Ortholog hit ratio analysis of assembled isotigs. A majority (63.8%) of all *G. bimaculatus* isotigs (black bars) have an ortholog hit ratio of ≥0.5 (blue arrowhead), and 40.0% have an ortholog hit ratio of ≥0.8 (red arrowhead). These values are higher than those obtained for the *O. fasciatus de novo* assembled transcriptome (grey bars) [11]. (B) Ortholog hit ratio analysis of unassembled singletons. As expected, singletons represent much smaller proportions of putative full-length transcripts. 6.3% of *G. bimaculatus* singletons (black) have an ortholog hit ratio of ≥0.5 (blue arrowhead), while 0.8% have an ortholog hit ratio of ≥0.8 (red arrowhead). As for the isotig analysis, these values are higher than those obtained for the *O. fasciatus de novo* assembled transcriptome (grey) [11].

To estimate the degree to which full-length transcripts might be predicted by the transcriptome, we determined the ortholog hit ratio [71] of all assembly products by comparing the BLAST results of the full assembly against the *Drosophila melanogaster* proteome. The ortholog-hit ratio is calculated as the ratio of the length of a transcriptome assembly product (isotig or singleton) and the full length of the corresponding transcript. Thus, a transcriptome sequence with an ortholog hit ratio of 1 would represent a full-length transcript. In the absence of a sequenced *G. bimaculatus* genome, for the purposes of this analysis we use the length of the cDNA of the best reciprocal BLAST hit against the *D. melanogaster* proteome as a proxy for the length of the corresponding transcript. For this reason, we do not claim that an ortholog hit ratio value indicates the true proportion f a full-length transcript, but rather that it is likely to do so. The full range of ortholog hit ratio values for isotigs and singletons is shown in Figure 4. Here we summarize two ortholog hit ratio parameters for both isotigs and singletons: the proportion of sequences with an ortholog hit ratio ≥0.5, and the proportion of sequences with an ortholog hit ratio ≥0.8. We found that 63.8% of *G. bimaculatus* isotigs likely represented at least 50% of putative full-length transcripts, and 40.0% of isotigs were likely at least 80% full length (Figure 4B). For singletons, 6.3% appeared to represent at least 50% of the predicted full-length transcript, and 0.9% were likely at least 80% full length (Figure 4B). Most ortholog hit ratio values were higher than those obtained for the *de novo* transcriptome assembly of another hemimetabolous insect, the milkweed bug *Oncopeltus fasciatus* [11] (Figure 4A, B). We suggest that this may be explained by the fact that the *G. bimaculatus de novo* transcriptome assembly contains transcript predictions of higher coverage and longer isotigs (N50 = 2,133 compared to 1,735 for *O. fasciatus* [11]) that are likely closer to predicted full-length transcript sequences, relative to the *O. fasciatus de novo* transcriptome assembly [11]. However, we cannot exclude the possibility that the higher ortholog hit ratios obtained with the *G. bimaculatus* transcriptome may be due to its greater sequence similarity with *D. melanogaster* relative to *O. fasciatus*. Genome sequences for the two hemimetabolous insects, and rigorous phylogenetic analysis for each predicted gene in both transcriptomes, would be necessary to resolve the origin of the ortholog hit ratio differences that we report here.

### Annotation using BLAST against the NCBI non-redundant protein database

All assembly products were compared with the NCBI non-redundant protein database (**nr**) using BLASTX. We found that 11,943 isotigs (55.52%) and 10,815 singletons (8.95%) were similar to at least one **nr** sequence with an E-value cutoff of 1e-5 (henceforth called “significant similarity”). The total number of unique BLAST hits against **nr** for all non-redundant assembly products (isotigs+singletons) was 19,874, which could correspond to the number of unique *G. bimaculatus* transcripts contained in our sample. The *G. bimaculatus* transcriptome contains more predicted transcripts than other orthopteran transcriptome projects to date (Table 1). This may be due to the high number of bp incorporated into our *de novo* assembly, which was generated from approximately two orders of magnitude more reads than previous Sanger-based orthopteran EST projects [72], [73], [74], [75], [76]. However, we note that even a recent Illumina-based locust transcriptome project that assembled over ten times as many base pairs as the *G. bimaculatus* transcriptome, predicted only 11,490 unique BLAST hits against **nr** [72]. This may be because the tissues we samples possessed a greater diversity of gene expression than those for the locust project, in which over 75% of the cDNA sequenced was obtained from a single nymphal stage [72]. Although we have used the de novo assembly method that was recommended as outperforming other assemblers in analysis of 454 pyrosequencing data [48], we cannot exclude the possibility that under-assembly of our transcriptome contributes to the high number of predicted transcripts

Since isogroups are groups of isotigs that are assembled from the same group of contigs, the isogroup number of 16,456 may represent the number of *G. bimaculatus* unique genes represented in the transcriptome. However, because by definition *de novo* assemblies cannot be compared with a sequenced genome, several issues limit our ability to estimate an accurate transcript or gene number for *G. bimaculatus* from these ovary and embryo transcriptome data alone.

The number of unique BLAST hits against **nr** (19,874) or isogroups (16,456) may overestimate the number of unique genes in our samples, because the assembly is likely to contain sequences derived from the same transcript but too far apart to share overlapping sequence; such sequences could not be assembled together into a single isotig and would therefore have been considered “different genes.” If such assembly products were derived from different regions of the same transcript and obtained distinct BLAST hits against **nr**, then these would be counted as two unique BLAST hits against **nr**. This limitation is an inevitable result of performing *de novo* assembly in the absence of a reference genome, and is unavoidable in the case of *G. bimaculatus* as no orthopteran genomes have yet been sequenced. Conversely, the number of unique BLAST hits against **nr** could underestimate the number of unique genes, because they cannot include those isotigs (9,569 = 44.5% of all isotigs) and singletons (109,990 = 91.0% of all singletons) that lacked significant BLAST hits against **nr**. Such sequences could represent non-coding sequences with no matches to the coding-region data contained in **nr**, or could lack sufficient similarity to known sequences. Finally, because our transcriptome libraries were prepared only from ovarian and embryonic tissue, it is unlikely to contain transcripts of all *G. bimaculatus* genes, many of which could be expressed exclusively postembryonically and/or in specific nymphal or adult tissue types. Determination of the total gene number for *G. bimaculatus* must therefore await complete genome sequencing.

**Figure 5:**
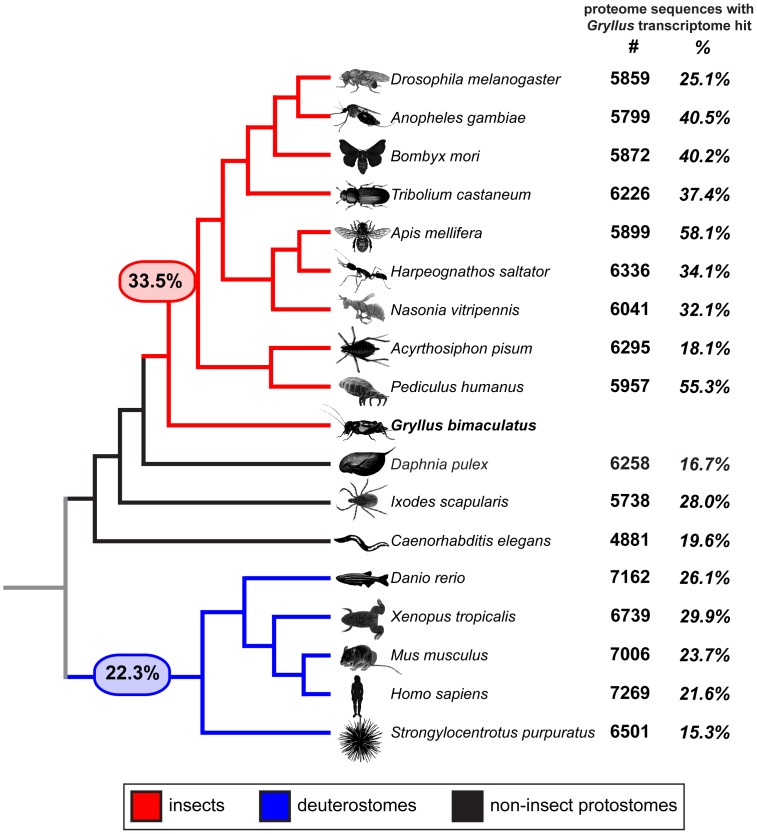
Phylogenetic comparison of proportion of known proteomes represented in the *G. bimaculatus de novo* assembled transcriptome. The number (bold) and percentage (bold italics) of proteome sequences with a putative *G. bimaculatus* ortholog in the *de novo* transcriptome assembly is shown for selected animals with sequenced genomes (based on top BLAST hit, E-value cutoff 1e-5). Proteomes were predicted from genome sequence sources as shown in Table S1. Numbers in large font in red and blue ovals indicate average proportion of sequences from all tested insect and deuterostome proteomes, respectively, represented in the *G. bimaculatus* transcriptome.

We wished to understand the relative similarities of the *G. bimaculatus* transcriptome sequences to those from other organisms. Specifically, we asked what proportion of genes found in sequenced animal genomes had putative orthologs in the *G. bimaculatus* transcriptome. To this end, we used BLAST to compare each non-redundant assembly product (E-value cutoff 1e-5) to the proteomes of several organisms with completely sequenced genomes (Table S1). We found that overall, 33.49% of the sequences contained in insect proteomes had matches in the *G. bimaculatus de novo* transcriptome assembly, compared to 22.28% of sequences from deuterostome proteomes (Figure 5). Within the insects, the proportion of hits to the *D. melanogaster* proteome was lower than the proportion of hits to most other insects. This may reflect the relatively greater divergence from a last common insect ancestor, as *D. melanogaster* belongs to the most derived insect order, the Diptera. However, we noted that the proportion of matches to some insect proteomes appeared unusually low given their phylogenetic relationship to Orthoptera. Specifically, only 18.1% of proteome sequences from the aphid *Acyrthosiphon pisum*, a hemimetabolous insect, had hits in the *G. bimaculatus* transcriptome, compared with an average of 36.1% across all holometabolous proteomes surveyed (Figure 5). This is consistent with the description of the *A. pisum* genome containing many unusual features relative to other insect genomes, including extensive gene family duplications and gene loss [6], [77], [78], [79]. The relatively high proportion of holometabolous proteome sequences with matches in the *G. bimaculatus* transcriptome suggests that these organisms may share more features derived from a last common insect ancestor than does *A. pisum*, and highlights the need for further genomic resources in the Hemimetabola. We caution that there are limitations to the biological information that can be derived from these comparisons, as not all animal genomes used for this analysis have comparable levels of coverage or annotation.

### Manual annotation of conserved developmental genes and members of signaling pathways

*G. bimaculatus* has been the subject of molecular embryology for over a decade, and as a result over 80 GenBank accessions are available (NCBI accessed 12 August 2012). We asked whether these genes were represented in our transcriptome, and found that 72.3% of them were present (60/83). Moreover, the transcriptome contributed to these accessions by extending their sequences by an average of 737 nucleotides per accession (205.0% on average across all 83 *G. bimaculatus* GenBank accessions) and in some cases by over 1,700% (Table S2). This shows that the *G. bimaculatus* transcriptome will be an extremely useful resource for continued research into the function and evolution of most previously cloned genes.

**Figure 6:**
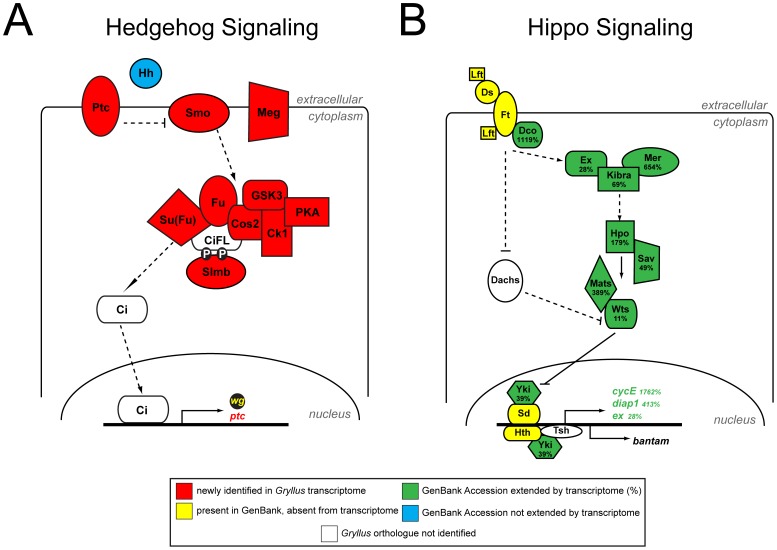
Sequence extension and gene discovery in the *G. bimaculatus* Hedgehog and Hippo pathways. (A) The *de novo* transcriptome assembly of *G. bimaculatus* newly identifies most members of the *hedgehog* pathway (red), from which only the *hedgehog* ligand (blue) was previously known (GenBank accession AB044709). (B) The transcriptome also adds significant sequence data to the fragments of many genes in the Hippo signaling pathway that had been previously identified (green). Seven genes of the known pathway were not identified in the transcriptome (yellow, white), two of which lack any sequence data in GenBank (white). GenBank accessions for previously identified sequences are as follows: *discs overgrown* (*dco*): AB443442; *expanded* (*ex*): AB378099; *warts* (*wts*): AB300574; *cyclin E* (*cycE*): AB378067; *hippo* (*hpo*): AB378070; *inhibitor of apoptosis protein* (*diap1*): AB378071; *mob as tumor suppressor* (*mats*): AB378072; *yorkie* (*yki*): AB378076; *scaffold protein salvador* (*sav*): AB378074; *Merlin* (*Mer*): AB378073; *Kibra:* DC445461.

To determine the transcriptome's utility as a source of new gene discovery, we searched for putative orthologs of the 1,168 *D. melanogaster* transcription factors catalogued in the FlyTF transcription factor database [80]. We found that 542 (46.4%) of them were present, based on the criterion of being the best reciprocal BLAST hit with a *D. melanogaster* sequence using an E-value cutoff of 1e-5 (Table S3). We also undertook manual annotation of 122 genes from seven conserved metazoan signaling pathways (Table S4), 261 genes involved in male and female gametogenesis in *D. melanogaster* (Table S5), and 24 additional genes with roles in maternal or zygotic embryonic patterning (Table S6). For the Notch [81], TGF-beta [82], Wnt [83], JAK/STAT [84], MAPK [85] and *hedgehog* [86] signaling pathways, most *G. bimaculatus* orthologs of these genes were previously unknown. Our transcriptome newly identified 66 genes participating in these signaling pathways (Table S4, Figure S2), including nearly all members besides the ligand of the *hedgehog* pathway (Figure 6A). In the case of the Hippo signaling pathway [87], for which most *G. bimaculatus* core kinase orthologs were already present in GenBank, the *G. bimaculatus de novo* transcriptome assembly increased the length of known sequences by an average of 323%, and by as much as 1,119% in the case of the *discs overgrown* (*dco*) gene (Figure 6B, Table S2).

### Automated annotation using the custom script “Gene Predictor” identifies 14,130 transcriptome sequences as putatively orthologous to *D. melanogaster* genes

**Figure 7:**
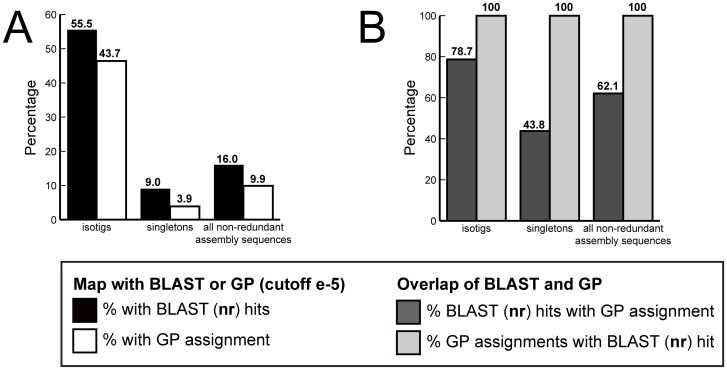
Automated annotation of the *G. bimaculatus de novo* transcriptome assembly using Gene Predictor. (A) Comparison of the proportion of non-redundant assembly sequences, isotigs and singletons that obtained a significant BLAST hit against **nr** (black bars), and those that were assigned a putative orthology by Gene Predictor (GP; white bars), based on the best reciprocal top BLAST hit with the *Drosophila melanogaster* proteome (see Table S1). (B) Comparison of the proportion of sequences with a significant BLAST hit in **nr** that also had a putative orthology assignment based on Gene Predictor (dark grey bars). All sequences assigned putative orthologs by Gene Predictor also had significant BLAST hits in **nr** (light grey bars).

Although manual annotation proved a highly effective way to identify developmental genes of interest in the *G. bimaculatus* transcriptome, it is not efficient at large scales. We therefore developed an automated annotation tool that uses the criterion of best reciprocal BLAST hit against the *D. melanogaster* proteome (E-value cutoff 1e-5) to propose putative orthologs for all assembly products of the transcriptome. This method is not qualitatively different from manual annotation using BLAST with a specific known sequence as a query, but rather simply automates the process of detecting a best reciprocal BLAST hit, which is a method of orthology assignment routinely employed as an annotation method in genomics studies using insect genomes [88], [89], [90]. Using this tool, called Gene Predictor (see Methods), we were able to assign putative orthologs to 43.7% of isotigs, very close to the proportion of isotigs (55.5%) with significant BLAST hits against **nr** (Figure 7A). Of the 60 known *G. bimaculatus* GenBank accessions that were identified in the transcriptome by manual annotation (Table S2), 52 have significant BLAST hits to a *D. melanogaster* gene (the remaining 8 genes have significant similarity only to non *D. melanogaster* sequences, as determined by BLAST against **nr**). Gene Predictor correctly identified 36 of these 52 genes (69.2%). Gene Predictor's failure to identify the remaining 16 genes (30.8%) means that while these genes do have significant BLAST hits in the *D. melanogaster* genome, they are more similar to a non-*D. melanogaster* gene, and are thus not the reciprocal best BLAST hit of any *D. melanogaster* gene.

These results suggest that for *de novo* insect transcriptome assemblies, Gene Predictor could be an efficient annotation tool, as it is nearly as effective as BLAST mapping against the large **nr** database, but is computationally much less intensive as it relies only on the *D. melanogaster* proteome of 23,361 predicted proteins. Relative to BLAST mapping against **nr**, Gene Predictor was more effective at suggesting orthologs for isotigs than for singletons (Figure 7A), likely due to the fact that isotigs are easier to map by any method as they contain more sequence data. Gene Predictor did not, however, assign orthologs to any assembly products that did not already have a significant BLAST hit in **nr** (Figure 7B), as expected since the *D. melanogaster* proteome is contained within **nr**. Conversely, not all assembly sequences with BLAST hits in **nr** obtained a significant hit with Gene Predictor (Figure 7B), indicating that some of the *G. bimaculatus* predicted transcripts share greater similarity to sequences other than those in the *D. melanogaster* proteome, or may represent genes that have been lost in *D. melanogaster*. The Gene Predictor scripts are freely available at http://www.extavourlab.com/protocols/index.html.

### Transcripts lacking significant BLAST hits against nr may encode functional protein domains

The majority (55.5%) of predicted transcripts retrieved a significant BLAST hit against the **nr** database (Figure 7A). This exceeds the proportion of *de novo* assembly products typically identifiable by BLAST mapping against **nr** [71], including the 43.4% and 29.5% of predicted transcripts mapped in this way from two *de novo* arthropod transcriptome assemblies that we previously constructed using similar methods to those described here [11], [49]. This may be due to the much higher read depth and coverage of the *G. bimaculatus* transcriptome, which to our knowledge is the largest *de novo* assembled transcriptome available for the Hemimetabola, and the largest 454-based transcriptome for any organism to date. Even this assembly, however, contains a large proportion (44.5%) of sequences of unknown identity. These sequences could represent contaminants of unknown origin, sequences that are too short to obtain significant hits to **nr** sequences, non-coding transcripts, non-coding portions of protein-coding transcripts, or clade- or species-specific transcripts that may be unidentifiable due to the paucity of orthopteran genomic data in GenBank. We believe that significant contaminants are unlikely, as less than one percent of all assembly products retrieved BLAST hits to prokaryote, fungal or plant sequences with an E-value cutoff of 1e-10.

**Figure 8:**
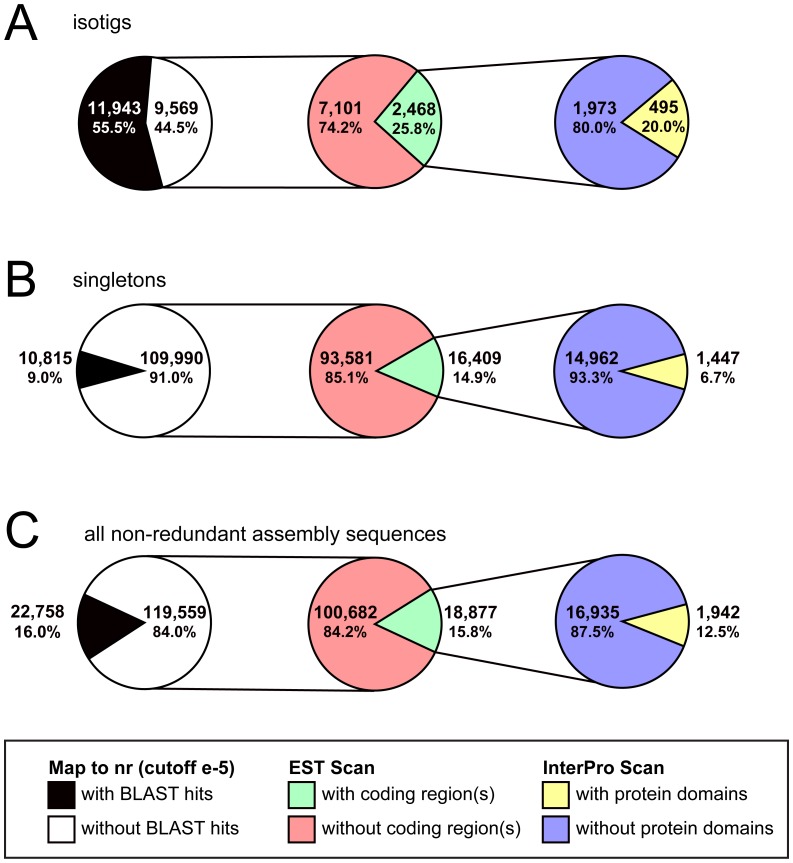
Coding region analysis of *G. bimaculatus de novo* transcriptome assembly sequences without significant BLAST hits in nr. Assembly products that failed to obtain significant BLAST hits in **nr** (white) were examined for the presence of coding regions (green) using EST Scan [52]. Assembly sequences thus predicted to contain coding regions were examined for the presence of known coding domains (yellow) using InterPro Scan [53], [54]. Results are shown separately for isotigs (A), singletons (B) and all non-redundant assembly products (C). See also Table 3.

We also compared the length (in nucleotides) of sequences with and without significant BLAST hits (Tables 3, 4), and found that unidentified isotigs were significantly shorter than isotigs with BLAST hits (Table 5). The difference was also significant for singletons (Tables 4, 5). This is consistent with the possibility that contig length may play a role in sequence recognizability, also observed with the low proportion of singletons with significant BLAST hits compared to isotigs (9.0% vs 55.5%; Figure 8A, B).

**Table 3:** Length parameters of isotigs according to BLAST annotation and predicted protein-coding status.

BLAST hit^1^/predicted protein coding status	Parameter	Value
**Significant hit against nr** 2 **^,^** 3	Maximum sequence length^4^	10865
	Minimum sequence length	91
	Median sequence length	1669.50
	Average sequence length	1927.98
**Significant hit against nr and contains predicted protein-coding region(s)**	Maximum sequence length	10865
	Minimum sequence length	168
	Median sequence length	1730.5
	Average sequence length	1997.42
	Maximum predicted peptide length^5^	2076
	Minimum predicted peptide length	11
	Median predicted peptide length	317.50
	Average predicted peptide length	386.82
**No significant hit against nr**	Maximum sequence length	6886
	Minimum sequence length	57
	Median sequence length	728.50
	Average sequence length	924.277
**No significant hit against nr and contains predicted protein-coding region(s)**	Maximum sequence length	6686
	Minimum sequence length	60
	Median sequence length	858.5
	Average sequence length	1130.16
	Maximum predicted peptide length	1710
	Minimum predicted peptide length	7
	Median predicted peptide length	144.5
	Average predicted peptide length	197.61
**All NRI** 6 **containing predicted protein-coding regions**	Maximum sequence length	10865
	Minimum sequence length	60
	Median sequence length	1544.50
	Average sequence length	1837.57
	Maximum predicted peptide length	2076
	Minimum predicted peptide length	7
	Median predicted peptide length	282.50
	Average predicted peptide length	351.95
**All NRI without predicted protein-coding regions**	Maximum sequence length	6677
	Minimum sequence length	57
	Median sequence length	708.50
	Average sequence length	878.27
**No significant hit against nr and significant hit against ** ***Locusta migratoria*** ** sequences** 7	Maximum sequence length	5287
	Minimum sequence length	124
	Median sequence length	1093.50
	Average sequence length	1358.21
	Maximum predicted peptide length	1710
	Minimum predicted peptide length	25
	Median predicted peptide length	244.50
	Average predicted peptide length	320.84
**No significant hit against nr and significant hit against ** ***Laupala kohalensis*** ** sequences** 8	Maximum sequence length	6677
	Minimum sequence length	62
	Median sequence length	1004.50
	Average sequence length	1304.64
	Maximum predicted peptide length	1710
	Minimum predicted peptide length	16
	Median predicted peptide length	248.50
	Average predicted peptide length	315.37

1 BLAST E-value cutoff is e-5 for all hits reported in this table.

2 **nr** = NCBI non-redundant database.

**Figure 9:**
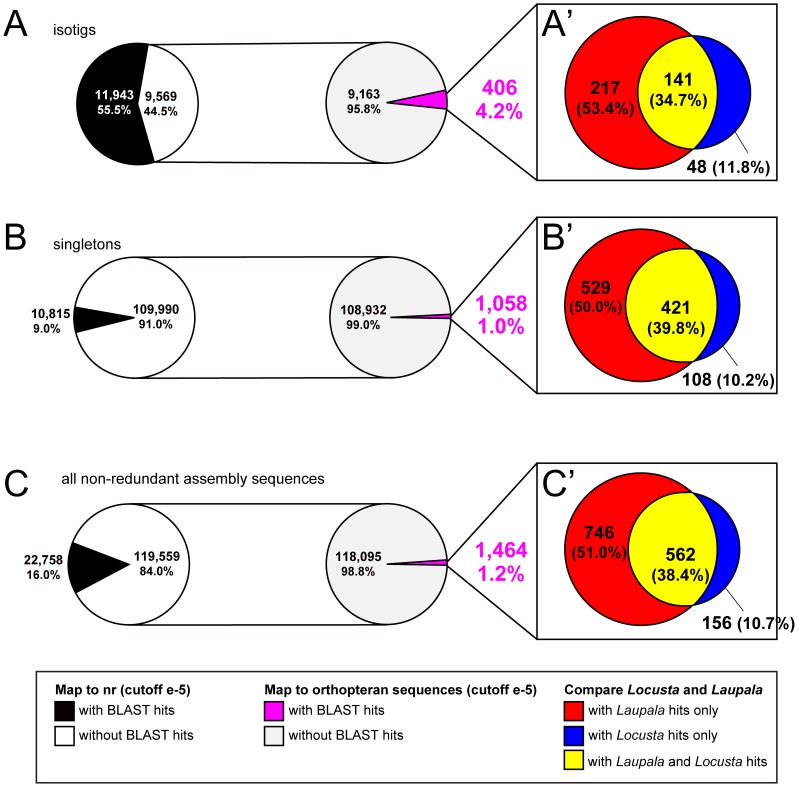
Comparison of sequences lacking significant BLAST hits to nr, with *Laupala kohalensis* and *Locusta m*igratoria databases. (A–C) Assembly products that failed to obtain significant BLAST hits to **nr** (white) were examined for significant similarity (magenta) to transcripts from at least one of *L. migratoria* or *L. kohalensis* [72], [73], [74], [75]. (A′–C′) Assembly sequences thus identified were parsed into sequences with significant hits among only *L. kohalensis* sequences (red), only *L. migratoria* sequences (blue), or both (yellow). Results are shown separately for isotigs (A, A′), singletons (B, A′) and all non-redundant assembly products (C, A′).

3 Numbers of sequences in each category are shown in Figure 9.

4 Sequence lengths are reported in base pairs.

5 Predicted peptide lengths are reported in amino acids.

6 NRI = all non-redundant isotigs regardless of BLAST results against **nr**.

7 *Locusta migratoria* sequences used for comparison are from [73], [74].

8 *Laupala kohalensis* sequences used for comparison are from [75].

**Table 4:** Length parameters of singletons according to BLAST annotation and predicted protein-coding status.

BLAST hit^1^/predicted protein coding status	Parameter	Value
**Significant hit against nr** 2,3	Maximum sequence length^4^	582
	Minimum sequence length	66
	Median sequence length	340.00
	Average sequence length	334.25
**Significant hit against nr and contains predicted protein-coding region(s)**	Maximum sequence length	574
	Minimum sequence length	68
	Median sequence length	343.5
	Average sequence length	337.54
	Maximum predicted peptide length^5^	192
	Minimum predicted peptide length	8
	Median predicted peptide length	103.50
	Average predicted peptide length	103.28
**No significant hit against nr**	Maximum sequence length	620
	Minimum sequence length	50
	Median sequence length	243.50
	Average sequence length	251.67
**No significant hit against nr and contains predicted protein-coding region(s)**	Maximum sequence length	586
	Minimum sequence length	50
	Median sequence length	231.5
	Average sequence length	243.16
	Maximum predicted peptide length	189
	Minimum predicted peptide length	5
	Median predicted peptide length	60.50
	Average predicted peptide length	65.02
**All NRS** 6 **containing predicted protein-coding region(s)**	Maximum sequence length	586
	Minimum sequence length	50
	Median sequence length	255.5
	Average sequence length	268.89
	Maximum predicted peptide length	192
	Minimum predicted peptide length	5
	Median predicted peptide length	71.5
	Average predicted peptide length	75.45
**All NRS without predicted protein-coding regions**	Maximum sequence length	620
	Minimum sequence length	50
	Median sequence length	249.50
	Average sequence length	255.51
**No significant hit against nr and significant hit against ** ***Locusta migratoria*** ** sequences** 7	Maximum sequence length	552
	Minimum sequence length	52
	Median sequence length	299
	Average sequence length	283.97
	Maximum predicted peptide length	176
	Minimum predicted peptide length	17
	Median predicted peptide length	74.50
	Average predicted peptide length	75.08
**No significant hit against nr and significant hit against ** ***Laupala kohalensis*** ** sequences** 8	Maximum sequence length	597
	Minimum sequence length	52
	Median sequence length	286.50
	Average sequence length	280.55
	Maximum predicted peptide length	188
	Minimum predicted peptide length	11
	Median predicted peptide length	77.5
	Average predicted peptide length	77.40

1 BLAST E-value cutoff is e-5 for all hits reported in this table.

2 **nr** = NCBI non-redundant database.

3 Numbers of sequences in each category are shown in Figure 9.

4 Sequence lengths are reported in base pairs.

5 Predicted peptide lengths are reported in amino acids.

6 NRS = all non-redundant singletons regardless of BLAST results against **nr**.

7 *Locusta migratoria* sequences used for comparison are from [73], [74].

8 *Laupala kohalensis* sequences used for comparison are from [75].

**Table 5:** Statistical comparison of isotig and singleton nucleotide sequence lengths according to BLAST annotation and predicted protein-coding status.

BLAST hit^1^/predicted protein coding status^2^	Significant hit against nr^2^	Significant hit against nr and contains predicted coding regions	No significant hit against nr	No significant hit against nr and contains predicted coding regions	All NRAS^3^ containing predicted protein-coding regions	All NRAS without predicted protein-coding regions	No significant hit against nr and significant hit against *Locusta migratoria* sequences	No significant hit against nr and significant hit against *Laupala kohalensis* sequences
**ISOTIGS** 4								
**Significant hit against nr**		0.9998	***	***	***	***	***	***
**Significant hit against nr and contains predicted coding regions**			1	***	1	1	1	1
**No significant hit against nr**				1	1	***	1	1
**No significant hit against nr and contains predicted coding regions**					***	1	***	***
**All NRAS containing predicted protein-coding regions**						***	***	***
**All NRAS without predicted protein-coding regions**							1	1
**No significant hit against nr and significant hit against ** ***Locusta migratoria*** ** sequences**								0.2268
**No significant hit against nr and significant hit against ** ***Laupala kohalensis*** ** sequences**								
**SINGLETONS**								
**Significant hit against nr**		0.9798	***	***	***	***	***	***
**Significant hit against nr and contains predicted coding regions**			1	***	1	1	1	1
**No significant hit against nr**				***	1	***	0.4208	1
**No significant hit against nr and contains predicted coding regions**					***	***	*	***
**All NRAS containing predicted protein-coding regions**						***	***	0.0969
**All NRAS without predicted protein-coding regions**							0.1358	0.9985
**No significant hit against nr and significant hit against ** ***Locusta migratoria*** ** sequences**								0.9967
**Significant hit against ** ***Laupala kohalensis*** ** sequences**								

Values shown are *p*≥0.05 value results of a Welch's t-test.

*** = *p*<0.0001;

* *p*<0.05.

1 BLAST E-value cutoff is e-5 for all hits reported in this table.

2 **nr** = NCBI non-redundant database.

3 NRAS = all non-redundant assembly products (isotigs or singletons) regardless of BLAST results against **nr**.

4 Numbers of sequences in each category are shown in Figure 9. Mean, median, maximum and minimum values for each category are shown in Tables 3 and 4.

To obtain additional biological information about sequences that failed to obtain significant BLAST hits against **nr**, we therefore applied EST Scan analysis to determine whether these sequences potentially encoded unknown proteins. EST Scan uses known differences in hexanucleotide usage between coding and non-coding regions to detect potential coding regions in DNA sequences, without requiring open reading frames [52]. We found that 2,468 (25.8%) unidentified isotigs and 16,409 (14.9%) unidentified singletons were predicted to contain protein-coding regions (Figure 8). Isotigs without predicted coding regions were significantly shorter than sequences with predicted coding regions (Tables 3, 5); the difference was also significant for singletons (Tables 4, 5). Previously unidentified isotigs that were protein-coding were significantly shorter that isotigs with significant BLAST hits, and encoded significantly fewer amino acids (Tables 3, 5, 6). This may mean that significant BLAST hits were not obtained for some of these sequences either because of insufficient contig lengths, or because they contained relatively less protein-coding content, or both. These observations demonstrate that although these 18,877 sequences are not significantly similar to known proteins in the NCBI nr database, they may nevertheless represent portions of coding rather than non-coding transcripts.

**Table 6:** Statistical comparison of isotig and singleton predicted coding sequence lengths according to BLAST annotation status.

BLAST hit^1^/predicted protein coding status^2^	Significant hit against nr^2^	No significant hit against nr	All NRAS^3^	No significant hit against nr and significant hit against *Locusta migratoria* sequences	No significant hit against nr and significant hit against *Laupala kohalensis* sequences
**ISOTIGS** 4					
**Significant hit against nr**		***	1	**	***
**No significant hit against nr**			***	1	1
**All NRAS**				*	0.0059
**No significant hit against nr and significant hit against ** ***Locusta migratoria*** ** sequences**					0.4052
**No significant hit against nr and significant hit against ** ***Laupala kohalensis*** ** sequences**					
**SINGLETONS**					
**Significant hit against nr**		***	1	***	***
**No significant hit against nr**			***	1	1
**All NRAS**				0.4091	0.9235
**No significant hit against nr and significant hit against ** ***Locusta migratoria*** ** sequences**					0.8685
**No significant hit against nr and significant hit against ** ***Laupala kohalensis*** ** sequences**					

Values shown are *p*≥0.05 value results of a Welch's t-test.

*** = *p*<0.0001;

** *p*<0.001;

* *p*<0.05.

1 BLAST E-value cutoff is e-5 for all hits reported in this table.

2 **nr** = NCBI non-redundant database.

3 NRAS = all non-redundant assembly products regardless of BLAST results against **nr**.

4 Numbers of sequences in each category are shown in Figure 9. Mean, median, maximum and minimum values for each category are shown in Tables 3 and 4.

We then used InterPro Scan [53], [54] to query predicted coding regions for predicted functional protein domains. InterPro Scan queries the InterPro consortium databases (ProDom [55], PRINTS [91], SMART [57], TIGRFAMs [58], Pfam [59], PROSITE [60], PIRSF [61], SUPERFAMILY [62], CATH [63], PANTHER [64], SignalPHMM [65], and Transmembrane [66]) for signatures of protein domains of known function. It also identifies evolutionarily conserved protein domains that are predicted to be functional based on their conservation but have no described molecular function to date, called Domains of Unknown Function (DUFs) [92]. This analysis revealed that of those protein-coding sequences of unknown identity, 495 (20.0%) isotigs and 1,447 (6.7%) singletons were predicted to contain functional protein domains. These results show that 1,942 sequences from the *de novo* transcriptome assembly that could not be identified based on BLAST against **nr** alone may nonetheless encode functional proteins present during *G. bimaculatus* oogenesis and embryogenesis.

### Taxonomic bias of the nr database can limit gene identification in *de novo* assembled transcriptomes

Because orthopteran sequence data are poorly represented in **nr**, we asked whether at least some of the *G. bimaculatus* transcriptome sequences that appeared to lack significant similarity to known genes might show similarity to sequences from other orthopterans available in the form of EST collections. To determine this, we compared the 9,569 isotigs (44.5% of all isotigs) and 109,990 singletons (91.0% of all singletons) from the *G. bimaculatus* transcriptome that lacked significant **nr** hits, with the EST collections for the orthopterans *L. migratoria* and *L. kohalensis*. *L. migratoria* of the suborder Caelifera (grasshoppers and locusts) is a migratory locust that is widespread throughout Asia, Africa, and Australasia [93], and is heavily studied due to its impact as an agricultural pest (see for example [94], [95]). The available sequence collections for this locust sampled transcripts from larval stages L4 and L5 [72], [73], [74], which is when transition between the solitary and gregarious (swarming) behavior of these locusts becomes irreversible [74], [96]. *L. kohalensis* belongs to the suborder Ensifera (katydids and crickets), and is a Hawaiian species that has been used extensively for studies of the physiology and evolution of speciation and acoustic preference (see for example [23], [97], [98]). The EST library available for this cricket contains sequences derived from transcripts of the larval central nervous system [75]. Because these data are derived from EST collections, they are available through GenBank but are not included in **nr**.

Using BLAST with an E-value cutoff of e-5, we found that the majority of previously unidentified *G. bimaculatus* transcriptome sequences also lacked significant similarity to *L. migratoria* or *L. kohalensis* sequences. This may be due to the difference in starting material for the libraries compared, as the *G. bimaculatus* transcriptome contains transcripts from ovaries and embryos, while the other two libraries represent exclusively post-embryonic transcripts, and the *L. kohalensis* library is further restricted only to transcripts from the nervous system. However, 406 isotigs (4.24%) and 1,058 singletons (0.96%) did display significant similarity (Figure 9A, B), suggesting that these transcripts could represent “orthopteroid” genes. However, we noted that sequences of both isotigs and singletons in this category contained significantly fewer nucleotides (Tables 3–5) and encoded significantly fewer amino acids on average (Tables 3, 4, 6) than transcriptome sequences with BLAST hits to **nr** (Tables 3–6). An alternative explanation for these apparent “orthopteroid” sequences is thus that these sequences, as well as their matches from *L kohalensis* and *L. migratoria*, might prove significantly similar to other sequences from **nr**, if their transcript sequences were longer.

Because Ensifera and Caelifera are believed to have diverged 300 Mya [5], we predicted that we would find greater similarity between sequences from the two crickets, than between *G. bimaculatus* and the locust. Accordingly, of the putative “orthopteroid sequences,” 746 (51.0%) *G. bimaculatus* sequences yielded hits exclusively to *L. kohalensis* sequences, compared to 156 (10.7%) sequences with exclusive hits among *L. migratoria* sequences (Figure 9C′). This likely reflects the closer phylogenetic relationship between the two crickets, which are both within the same family of Gryllidae.

### Putative orthopteroid-specific sequences contain a high proportion of predicted protein coding domains of unknown function (DUFs)

Finally, we asked whether these “orthopteroid sequences” shared any characteristics that might aid in understanding their putative clade-specific functions. We used InterPro Scan [53] to determine the distribution of recognizable protein domains among transcriptome sequences with significant *L. kohalensis* or *L. migratoria* hits, and compared them with those of all transcriptome sequences with significant BLAST hits to **nr**. We found that the number of distinct domains was similar for *L. kohalensis*-like sequences (77 different protein domains) and all other transcriptome sequences with significant BLAST hits (83 different protein domains), but considerably lower for *L. migratoria*-like sequences (55 different protein domains). Given the small number of sequences examined here (Figure 9C), this is unlikely to represent true differences in protein type between the three datasets.

**Figure 10:**
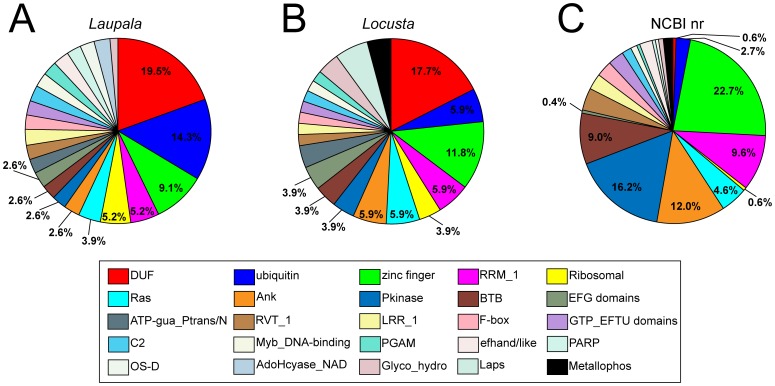
Principal protein domain composition of *G. bimaculatus* transcriptome sequences with highest similarity to *Laupala kohalensis* or *Locusta migratoria* sequences. Relative proportions of the top 25 protein domains coded by *G. bimaculatus* transcriptome sequences with significant similarity to sequences from *L. kohalensis* (A), *L. migratoria* (B), or sequences from **nr** (C). Protein domain nomenclature from Pfam [102] as follows: AdoHcyase_NAD: PF00670; Ank: PF00023; ATP-gua_Ptrans/N: PF02807; BTB/POZ: PF00651; C2: PF00168; DUF (combined): n/a; EFG domains (combined): n/a; efhand/like: PF09279; F-box: PF00646; Glyco_hydro (combined): n/a; GTP_EFTU domains: PF00009; Laps: PF10169; LRR_1: PF00560; Metallophos: PF00149; Myb_DNA-binding (combined): n/a; OS-D: PF03392; PARP: PF00644; PGAMP: PF07644; Pkinase: PF00069; Ras: PF00071; Ribosomal (combined): n/a; RRM_1: PF00076; RVT_1: PF00078; ubiquitin: PF00240; zinc finger (combined): n/a. “Combined” indicates that multiple Pfam accessions are combined.

However, the datasets differed strikingly in the relative proportions of different protein domains encoded. Considering the top 25 most frequently represented protein domains within each dataset, the most abundant domains in both orthopteran-like groups were domains of unknown function (DUFs, 18.8% of both orthopteran matches combined), followed by ubiquitin family domains (Pfam PF00240, 10.9%), zinc finger domains (multiple Pfam categories combined, 10.2%), and RNA recognition motifs (Pfam PF00076, 5.5%) (Figure 10A, B). In contrast, transcriptome sequences with significant BLAST hits to **nr** encoded proteins principally containing zinc finger domains (multiple Pfam categories combined, 22.7%), protein kinase domains (Pfam 00069, 16.2%), and ankyrin repeat domains (Pfam PF00023, 12.0%), followed by RNA recognition motifs (Pfam PF00076, 9.6%) and BTB/POZ domains (Pfam PF00651, 9.0%) (Figure 10C). These differing proportions of predicted protein domains between orthopteran-matched and **nr**-matched *G. bimaculatus* sequences were observed even when all predicted protein domains were considered (Figure S3). We speculate that the “orthopteroid-like” proteins predicted to be present in the *G. bimaculatus* transcriptome might share greater functional similarity with orthopteran proteins than with proteins from other organisms represented in **nr**. Moreover, the high proportion of DUFs predicted in these “orthopteroid-like” proteins may mean that some of these DUFs serve clade-specific functions. The specific roles of these genes in *G. bimaculatus* and other orthopterans are currently unknown, and will require functional genetic testing to be elucidated. However, the present analysis demonstrates that even for *de novo* assembled transcriptome sequences that are not easily identifiable based on GenBank comparisons, it may be possible to extract potentially meaningful biological and evolutionary information, and with further refinement, perhaps even to define new or clade-specific DUFs as candidates for future functional testing.

### Creation of a searchable database to house arthropod *de novo* assembled transcriptomes

The volume of high-throughput transcriptome data available for all organisms is rapidly increasing, but many of these datasets are not publicly available in an easily searchable format. The NCBI Short Read Archive [99] provides a repository for raw read data from transcriptome projects, but a searchable interface for *de novo* assembled transcriptomes that do not have an associated genome sequence or previously developed community web interface is lacking. Like EST collections, transcriptome assemblies can be made public through the NCBI Transcriptome Shotgun Assembly Sequence Database (TSA: http://www.ncbi.nlm.nih.gov/genbank/tsa), but annotation of these data is not required, and they are not included in **nr**. To maximize the public utility of our data, we therefore created a searchable database that facilitates access to the annotated *G. bimaculatus de novo* assembled transcriptome reported here. The Assembled Searchable Giant Arthropod Read Database (ASGARD) includes all **nr** BLAST, manual annotation, and Gene Predictor annotation results for the *G. bimaculatus* transcriptome. Details of the design and database schema of ASGARD have been previously described [51]. This database also contains two additional *de novo* assembled transcriptomes that we constructed previously, for the milkweed bug *Oncopeltus fasciatus* [11] and the amphipod crustacean *Parhyale hawaiensis* [49]. The *O. fasciatus* transcriptome, which was originally assembled with Newbler v2.3 [11], was re-assembled with Newbler 2.5, which was used to assemble the *P. hawaiensis* and *G. bimaculatus* transcriptomes. Complete updated assembly files in FASTA format for all three transcriptomes can be downloaded via ASGARD. We also processed the *O. fasciatus* and *P. hawaiensis* transcriptomes with the EST Scan, InterPro Scan, and the Gene Predictor script, so that they could be searched in the same way as the *G. bimaculatus* transcriptome. ASGARD allows users to search these *de novo* assembled transcriptomes in four ways: (1) for putative orthologs to known *D. melanogaster* genes (based on Gene Predictor results); (2) by searching the text of the top 50 significant BLAST hits for the name of any gene of interest (based on **nr** BLAST mapping results); (3) by searching for transcripts with a given GO term assignment; and (4) by read name if the unique identifier of a given assembly product is known (this information is provided in the results of the previous three searches). All search result output pages allow users to view and download the nucleotide sequences of matching assembly products, the pre-computed results of a BLAST search of that sequence against **nr** (E-value cutoff 1e-5), their predicted translation products if applicable (determined using EST Scan), and any predicted functional protein domains (determined using InterPro Scan). Finally, ASGARD also contains a BLAST interface that allows users to search any or all transcriptomes using the BLASTN, TBLASTN or TBLASTX algorithms. ASGARD is available at http://asgard.rc.fas.harvard.edu.

## Supporting Information

Figure S1 **Comparison of read lengths from** ***de novo*** **assembly of the** ***G. bimaculatus*** **transcriptome.** (A) Distribution of read lengths before (black) and after (blue) trimming to remove low quality reads (see text for details). (B) Distribution of trimmed read lengths before (blue) and after (red) assembly with Newbler v2.5. The assembly yielded assembled reads of over 10,000 bp. (C) Distribution of read lengths of the shortest assembled (red) and raw (blue) reads.
(TIF)

Figure S2 **Schematics of conserved metazoan signal transduction pathways showing components identified in the** ***G. bimaculatus*** **transcriptome.** BLAST was used to search for signaling pathway genes in the *G. bimaculatus* transcriptome (see Table S4); genes with newly identified putative orthologs are indicated in red. Genes outlined in grey with grey typeface indicate genes without *D. melanogaster* homologs. Pathway schematics are modified from KEGG pathway model images (http://www.genome.jp/kegg/kegg1.html). (A) Notch pathway. (B) TGF-βeta pathway. (C) Wnt pathway. (D) Janus Kinase (JAK)-signal transducer and activator of transcription (STAT) pathway. (E) Mitogen-activated protein Kinase (MAPK) pathway.
(TIF)

Figure S3 **Complete protein domain composition of** ***G. bimaculatus*** **transcriptome sequences with highest similarity to** ***Laupala kohalensis*** **or** ***Locusta migratoria*** **sequences.** Relative proportions of all protein domains coded by *G. bimaculatus* transcriptome sequences with significant similarity to sequences from *L. kohalensis* (A), *L. migratoria* (B), or sequences from **nr** (C). Protein domain nomenclature from Pfam [102] and SMART [103] databases as follows: 5_nucleotid_C: PF2872; Abhydrolase_1: PF00561; adh_short: PF00106; ADK: OF00406; AdoHcyase_NAD: PF00670; Amidohydro_1: PF01979; Ank: PF00023; AP_endonuc_2_N: PF07582; Asparaginase_2: PF01112; ATP-gua_Ptrans/N: PF02807; BAH: PF01426; BTB/POZ: PF00651; Btz: SM 01044; bZIP_2: PF07716; C2: PF00168; CBM_14: PF01607; COesterase: PF00135; Cyclin_N: PF00134; Cys_Met_Meta_PP: PF01053; DEAD: PF00270; DUF (combined): n/a; EFG domains (combined): n/a; efhand/like: PF09279; eIF-5_eIF-2B: PF01873; ELM2: PF01448; ELO: PF01151; EMP70: PF02990; ETF_alpha: PF00766; Exo_endo_phos: PF03372; F-box: PF00646; fn3: PF00041; G-patch: PF01858; GATA: PF00320; GCV_H: PF01597; GHMP_kinases_N: PF00288; Glyco_hydro (combined): n/a; GTP_EFTU domains: PF00009; HECT: PF00632; Hemocyanin_N: PF03722; HSP90: PF00183; IF-2B: PF01008; IPP-2: PF04979; JHBP: PF06585; Laps: PF10169; Ldl_recept_a: PF00057; Lectin_C: PF00059; LRR_1: PF00560; MA3: PF00560; MADF_DNA_bdg: PF10545; MAP65_ASE1: PF03999; Metallophos: PF00149; MIF4G: PF02854; Myb_DNA-binding (combined): n/a; NAC: PF01849; NAP: PF00956; NDUF_B8: PF05821; NIPSNAP: PF07978; Nucleoplasmin: PF03066; OS-D: PF03392; p450: PF00067; PABP: PF00658; PARP: PF00644; Peptidase_M17: PF00883; PGAMP: PF07644; PH: PF00169; PI-PLC-X/Y: PF00378/8; Pkinase: PF00069; PTPS: PF01242; Ras: PF00071; Ribophorin_I: PF04597; Ribosomal (combined): n/a; RNA_pol_A_bac: PF01000; RnaseH: PF00075; RRM_1: PF00076; RVT_1: PF00078; SAM_1: PF00536; Sedlin_N: PF04628; Serpin: PF00079; SH2: PF00017; SH3_1: PF00018; SNase: PF00565; Stathmin: PF008310; Synaptobrevin: PF00957; Thioredoxin: PF00085; Thymosin: PF01290; TRAP-gamma: PF07074; TRM: PF02005; TUDOR: PF00567; ubiquitin: PF00240; W2: PF02020; WD40: PF00400; zinc finger (combined): n/a. “Combined” indicates that multiple Pfam accessions are combined.
(TIF)

Table S1 **Sources of proteome sequences from animals with sequenced genomes used for comparison with the** ***G. bimaculatus de novo*** **transcriptome assembly.** Sequences were used for ortholog hit ratio analyses (Figure 3) and phylogenetic comparisons of proportion of proteome sequences for which putative *G. bimaculatus* orthologs were found (Figure 4).
(PDF)

Table S2 **Contribution of the** ***G. bimaculatus*** **transcriptome to GenBank accessions.** Sequences of *G. bimaculatus* developmental genes from GenBank were used as a query to BLAST the *de novo* transcriptome assembly. Matches in the transcriptome were found among both assembled reads and singletons.
(PDF)

Table S3 **FlyTF transcription factor orthologs identified in the** ***G. bimaculatus*** **transcriptome.** BLAST (E-cutoff 1e-5) was used to search the *G. bimaculatus* transcriptome for orthologs to the transcription factors belonging to the FlyTF database [80].
(PDF)

Table S4 **Selected signaling pathway genes identified in the** ***G. bimaculatus*** **transcriptome.** Hit ID indicates if gene hits were found assembled reads (A) or singletons (S). Length (range) indicates the shortest and longest A or S hit sequences for each gene. Query organism was *D. melanogaster* for all cases.
(PDF)

Table S5 **Selected gametogenesis genes identified in the** ***G. bimaculatus*** **transcriptome.** Hit ID indicates if gene hits found were assembled reads (A) or singletons (S). Length (range) indicates the shortest and longest A or S hit sequences for each gene. Groups of hits of a given color indicate transcriptome sequences that mapped to the same overlapping region of the BLAST target (putative SNPs or isoforms); hits of different colors indicate transcriptome sequences that map to different, non-overlapping regions of the BLAST target. Query organism was *D. melanogaster* for all cases.
(PDF)

Table S6 **Selected developmental process genes identified in the** ***G. bimaculatus de novo*** **transcriptome assembly.** Hit ID indicates if gene hits found were assembled reads (A) or singletons (S). Length (range) indicates the shortest and longest A or S hit sequences for each gene. Groups of hits of a given color indicate transcriptome sequences that mapped to the same overlapping region of the BLAST target (putative SNPs or isoforms); hits of different colors indicate transcriptome sequences that map to different, non-overlapping regions of the BLAST target. Query organism was *D. melanogaster* for all cases.
(PDF)
