# Supplementary figures and images for: Developmental Gene Discovery in a Hemimetabolous Insect: De Novo Assembly and Annotation of a Transcriptome for the Cricket Gryllus bimaculatus
Source: PLoS One. 2013 May 6;8(5):e61479. doi: 10.1371/journal.pone.0061479 (PMC3646015; doi:10.1371/journal.pone.0061479)

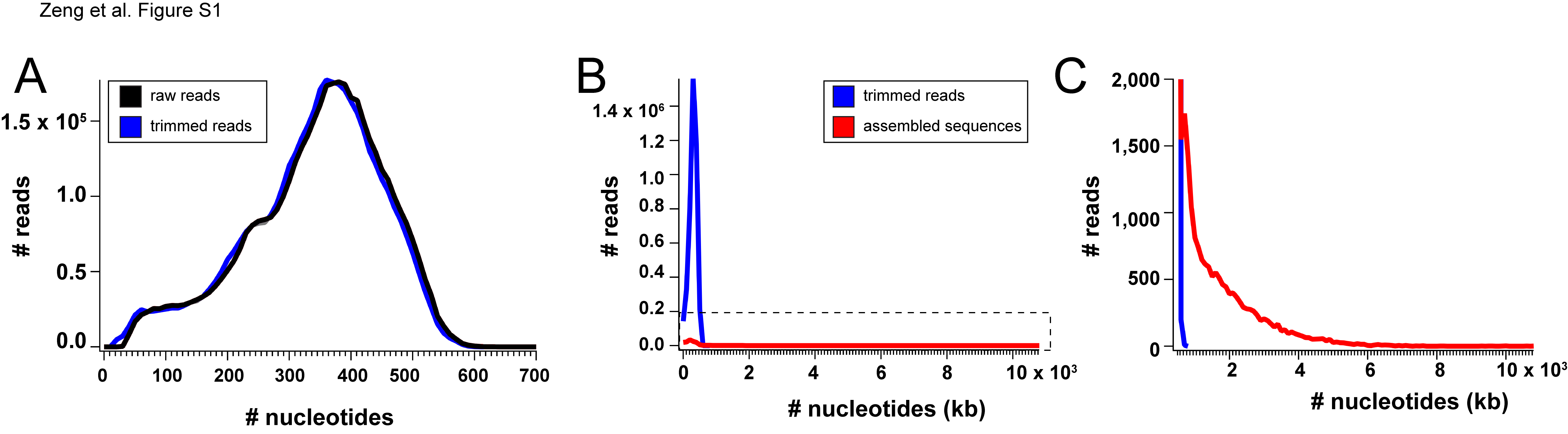

Supplement: Figure S1 — Comparison of read lengths from de novo assembly of the G. bimaculatus transcriptome. (A) Distribution of read lengths before (black) and after (blue) trimming to remove low quality reads (see text for details). (B) Distribution of trimmed read lengths before (blue) and after (red) assembly with Newbler v2.5. The assembly yielded assembled reads of over 10,000 bp. (C) Distribution of read lengths of the shortest assembled (red) and raw (blue) reads. (TIF) [file pone.0061479.s001.tif]

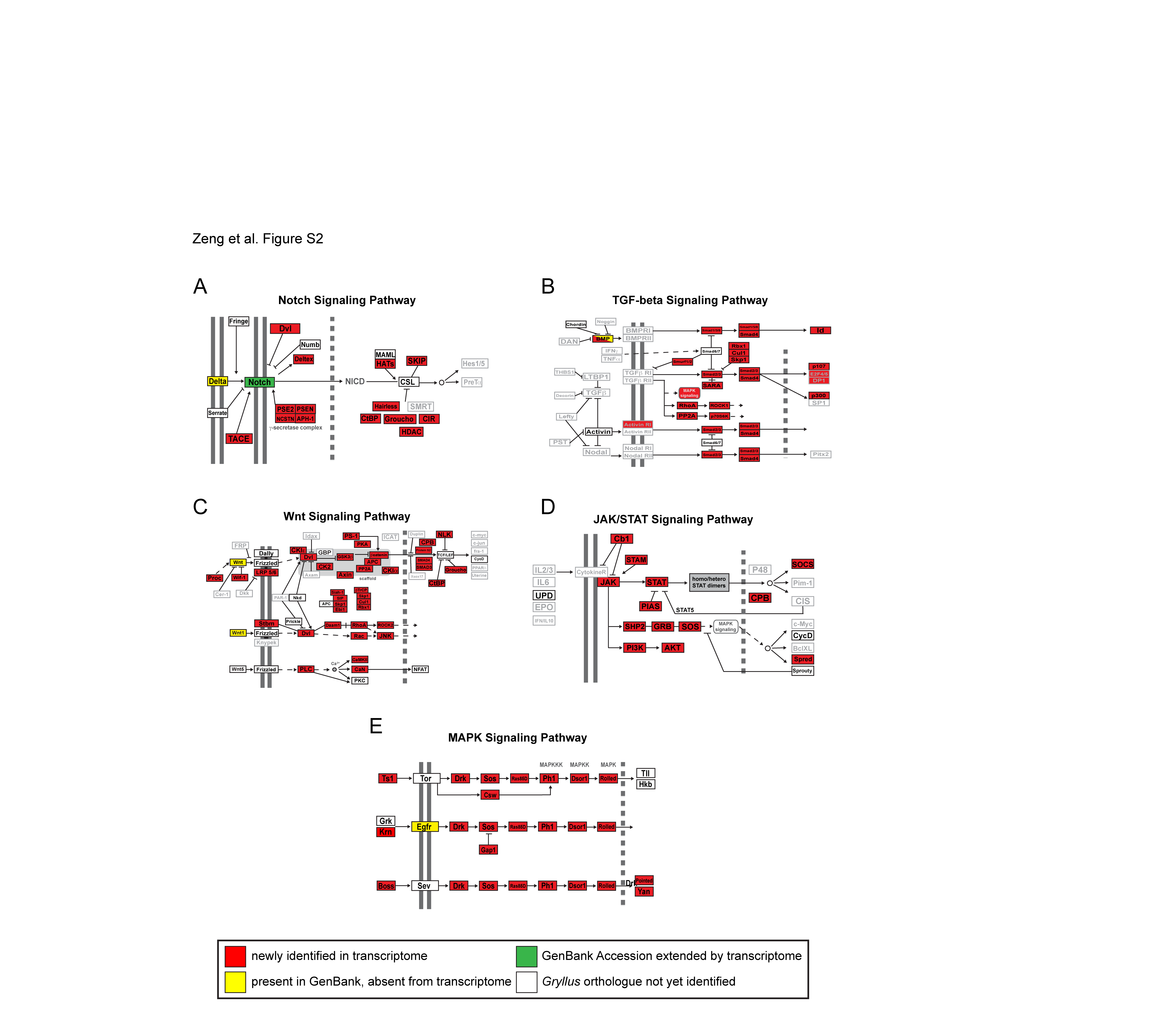

Supplement: Figure S2 — Schematics of conserved metazoan signal transduction pathways showing components identified in the G. bimaculatus transcriptome. BLAST was used to search for signaling pathway genes in the G. bimaculatus transcriptome (see Table S4); genes with newly identified putative orthologs are indicated in red. Genes outlined in grey with grey typeface indicate genes without D. melanogaster homologs. Pathway schematics are modified from KEGG pathway model images (http://www.genome.jp/kegg/kegg1.html). (A) Notch pathway. (B) TGF-βeta pathway. (C) Wnt pathway. (D) Janus Kinase (JAK)-signal transducer and activator of transcription (STAT) pathway. (E) Mitogen-activated protein Kinase (MAPK) pathway. (TIF) [file pone.0061479.s002.tif]

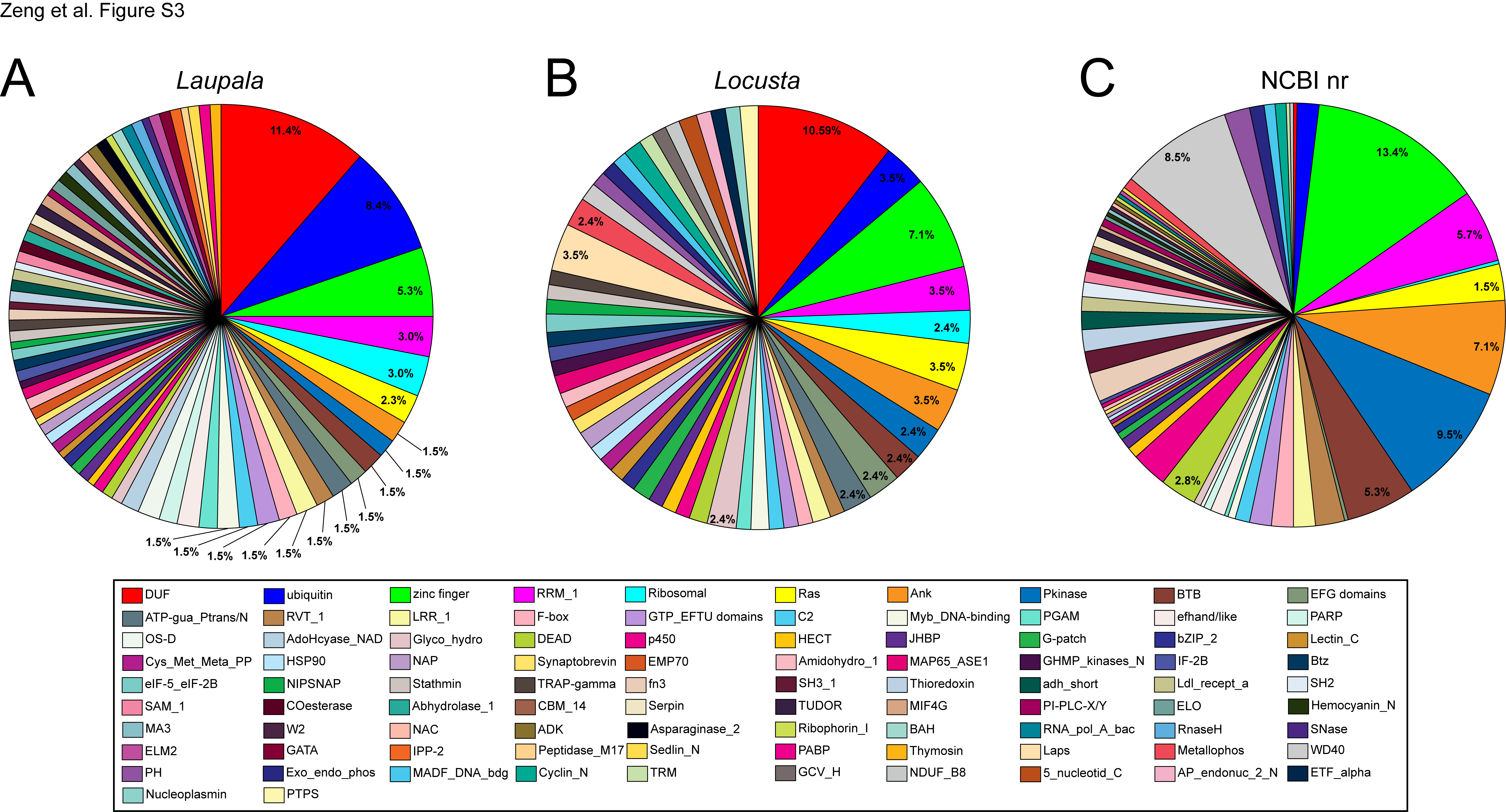

Supplement: Figure S3 — Complete protein domain composition of G. bimaculatus transcriptome sequences with highest similarity to Laupala kohalensis or Locusta migratoria sequences. Relative proportions of all protein domains coded by G. bimaculatus transcriptome sequences with significant similarity to sequences from L. kohalensis (A), L. migratoria (B), or sequences from nr (C). Protein domain nomenclature from Pfam [102] and SMART [103] databases as follows: 5_nucleotid_C: PF2872; Abhydrolase_1: PF00561; adh_short: PF00106; ADK: OF00406; AdoHcyase_NAD: PF00670; Amidohydro_1: PF01979; Ank: PF00023; AP_endonuc_2_N: PF07582; Asparaginase_2: PF01112; ATP-gua_Ptrans/N: PF02807; BAH: PF01426; BTB/POZ: PF00651; Btz: SM 01044; bZIP_2: PF07716; C2: PF00168; CBM_14: PF01607; COesterase: PF00135; Cyclin_N: PF00134; Cys_Met_Meta_PP: PF01053; DEAD: PF00270; DUF (combined): n/a; EFG domains (combined): n/a; efhand/like: PF09279; eIF-5_eIF-2B: PF01873; ELM2: PF01448; ELO: PF01151; EMP70: PF02990; ETF_alpha: PF00766; Exo_endo_phos: PF03372; F-box: PF00646; fn3: PF00041; G-patch: PF01858; GATA: PF00320; GCV_H: PF01597; GHMP_kinases_N: PF00288; Glyco_hydro (combined): n/a; GTP_EFTU domains: PF00009; HECT: PF00632; Hemocyanin_N: PF03722; HSP90: PF00183; IF-2B: PF01008; IPP-2: PF04979; JHBP: PF06585; Laps: PF10169; Ldl_recept_a: PF00057; Lectin_C: PF00059; LRR_1: PF00560; MA3: PF00560; MADF_DNA_bdg: PF10545; MAP65_ASE1: PF03999; Metallophos: PF00149; MIF4G: PF02854; Myb_DNA-binding (combined): n/a; NAC: PF01849; NAP: PF00956; NDUF_B8: PF05821; NIPSNAP: PF07978; Nucleoplasmin: PF03066; OS-D: PF03392; p450: PF00067; PABP: PF00658; PARP: PF00644; Peptidase_M17: PF00883; PGAMP: PF07644; PH: PF00169; PI-PLC-X/Y: PF00378/8; Pkinase: PF00069; PTPS: PF01242; Ras: PF00071; Ribophorin_I: PF04597; Ribosomal (combined): n/a; RNA_pol_A_bac: PF01000; RnaseH: PF00075; RRM_1: PF00076; RVT_1: PF00078; SAM_1: PF00536; Sedlin_N: PF04628; Serpin: PF00079; SH2: PF00017; SH3_1: PF00018; SNase: PF00565; Stathmin: PF008310; Synapt [file pone.0061479.s003.tif]
