# Supplementary material for: Developmental Gene Discovery in a Hemimetabolous Insect: De Novo Assembly and Annotation of a Transcriptome for the Cricket Gryllus bimaculatus
Source: PLoS One. 2013 May 6;8(5):e61479. doi: 10.1371/journal.pone.0061479 (PMC3646015; doi:10.1371/journal.pone.0061479)
Supplement: Table S1 — Sources of proteome sequences from animals with sequenced genomes used for comparison with the G. bimaculatus de novo transcriptome assembly. Sequences were used for ortholog hit ratio analyses (Figure 3) and phylogenetic comparisons of proportion of proteome sequences for which putative G. bimaculatus orthologs were found (Figure 4). (PDF) [file pone.0061479.s004.pdf]

Sources of animal proteome data used for phylogenetic comparisons of *G. bimaculatus* transcriptome sequence matches

**Species**	**Proteome Source**	**Dowload Date**
Apis mellifera	http://hymenopteragenome.org/drupal/sites/hymenopteragenome.org.beebase/files/data/Amel_release1_OGS_pep.fa.gz	25Mar11
Pediculus humanus	ftp://ftp.vectorbase.org/public_data/organism_data/phumanus/Geneset/pediculus_humanus_PhumU1.2.fa.gz	25Mar11
Anopheles gambiae	ftp://ftp.vectorbase.org/public_data/organism_data/agambiae/Geneset/anopheles_gambiae_AgamP3.6.fa.gz	25Mar11
Bombyx mori	ftp://silkdb.org/pub/current/Gene/silkpep.fa.gz	25Mar11
Laupala kohalensis ESTs	http://combio.dfci.harvard.edu	4May11
Locusta migratoria ESTs	http://locustdb.genomics.org.cn/download/Locust_EST.zip	4May11
Tribolium castaneum	ftp://bioinformatics.ksu.edu/pub/BeetleBase/3.0/Sequences/Tribolium_Official_Gene_Sequences/peptide.fa	25Mar11
Camponotus floridanus	http://hymenopteragenome.org/drupal/sites/hymenopteragenome.org.camponotus/files/data/cflo_v3.3.fa	25Mar11
Saccharomyces cerevisiae	http://downloads.yeastgenome.org/sequence/S288C_reference/orf_protein/orf_trans_all.fasta.gz	4May11
Aedes aegypti	ftp://ftp.vectorbase.org/public_data/organism_data/aaegypti/Geneset/aedes_aegypti_AaegL1.2.fa.gz	25Mar11
Harpegnathos saltator	http://hymenopteragenome.org/drupal/sites/hymenopteragenome.org.harpegnathos/files/data/hsal_v3.3.fa.gz	4May11
Culex quinquefasciatus	ftp://ftp.vectorbase.org/public_data/organism_data/cquinquefasciatus/Geneset/culex_quinquefasciatus_CpipJ1.2.fa.gz	25Mar11
Gallus gallus	ftp://ftp.ncbi.nih.gov/genomes/Gallus_gallus/protein/Gnomon_prot.fsa.gz	4May11
Nasonia vitripennis	http://genomes.arc.georgetown.edu/nasonia/nasonia_genome_consortium/data/Nvit_OGSv1.2_pep.fa.gz	25Mar11
Xenopus tropicalis	ftp://ftp.ncbi.nih.gov/refseq/X_tropicalis/mRNA_Prot/frog.protein.faa.gz	4May11
Ixodes scapularis	ftp://ftp.vectorbase.org/public_data/organism_data/iscapularis/Geneset/ixodes_scapularis_IscaW1.1.fa.gz	4May11
Danio rerio	ftp://ftp.ncbi.nih.gov/genomes/D_rerio/protein/Gnomon_prot.fsa.gz	4May11
Drosophila melanogaster	ftp://ftp.flybase.net/genomes/Drosophila_melanogaster/current/fasta/dmel-all-translation-r5.35.fasta.gz	25Mar11
Mus musculus	ftp://ftp.ncbi.nih.gov/genomes/M_musculus/protein/Gnomon_prot.fsa.gz	4May11
Homo sapiens	ftp://ftp.ncbi.nih.gov/refseq/H_sapiens/mRNA_Prot/human.protein.faa.gz	4May11
Caenorhabditis elegans	ftp://ftp.wormbase.org/pub/wormbase/species/c_elegans/sequence/protein/c_elegans.current.protein.fa.gz	4May11
Acyrthosiphon pisum	http://arthropods.eugenes.org/aphid/data/geneset1/ACYPIprot.fa.gz	25Mar11
Daphnia pulex	ftp://iubio.bio.indiana.edu/daphnia/genome/Daphnia_pulex/current/fasta/dpulex-all-translation-jgi060905.fasta.gz	11Dec11
Strongylocentrotus purpuratus	ftp://ftp.ncbi.nih.gov/genomes/Strongylocentrotus_purpuratus/protein/Gnomon_prot.fsa.gz	4May11
Escherichia coli	ftp://ftp.ncbi.nih.gov/genomes/Bacteria/Escherichia_coli_K_12_substr__DH10B_uid58979/NC_010473.faa	4May11
