# Supplementary material for: Developmental Gene Discovery in a Hemimetabolous Insect: De Novo Assembly and Annotation of a Transcriptome for the Cricket Gryllus bimaculatus
Source: PLoS One. 2013 May 6;8(5):e61479. doi: 10.1371/journal.pone.0061479 (PMC3646015; doi:10.1371/journal.pone.0061479)
Supplement: Table S2 — Contribution of the G. bimaculatus transcriptome to GenBank accessions. Sequences of G. bimaculatus developmental genes from GenBank were used as a query to BLAST the de novo transcriptome assembly. Matches in the transcriptome were found among both assembled reads and singletons. (PDF) [file pone.0061479.s005.pdf]

Table S2

Contribution of the *G. bimaculatus* transcriptome to existing *G. bimaculatus* GenBank accessions.

Gene Name	Accession #	Accession Sequence Length (nt)	total # nt added by transcriptome	# 5' nt added by transcriptome	# 3' nt added by transcriptome	% Accession lengthened by transcriptome	Transcriptome Read Name	Consensus Region	Query Location
14-3-3epsilon	AB443441	460	25	25	0	5%	GE8SX9M02IK8UO	1-365	1-367
		460	111	0	111	24%	GFCP6CO02GWC4H	425-112	151-460
14-3-3zeta	AB443440	438	2605	19	2586	595%	isotig03712	2597-3034	full
		438	2605	19	2586	595%	isotig03711	2597-2962	73-438
16s ribosomal	AF248685	498	207	207	0	42%	GE8SX9M02GQ4TQ	208-423, 493-533	1-215, 280-331
		498	120	0	120	24%	GE8SX9M01ELV4A	121-329	289-498
18S ribosomal	AF514548	1021	175	175	0	17%	isotig14176	176-799	1-627
		1021	938	0	938	92%	contig11156	939-1037	922-1021
28s ribosomal	EU878290	726	90	90	0	12%	isotig07604	91-377	1-287
		726	283	283	0	39%	isotig07603	284-570	1-287
		726	64	0	64	9%	isotig20138	65-144	645-724
abdominal-A	AB194277	868	309	309	0	36%	GFCP6CO01AM666	310-375, 386-458	708-780, 790-854
accessory gland protein	DQ630916	570	0	0	0	0%	GE8SX9M01BFM7Q	full	19-246
actin	AB087882	1290	0	0	0	0%	GFJY65E02JJW4Q	3-391	453-522, 874-1024, 1106-1275
aristaless	AB071147	2857	0	0	0	0%	GFCP6CO01BE1VK	full	2587-2831
armadillo protein	AB109212	3836	160	160	0	4%	isotig05341	161-2579, 2604-3966	11-2428, 2453-3811
beta-actin	DQ630919	210	203	138	65	97%	GFJY65E02ICT38	66-274	1-210
chico	AB370294	440	37	37	0	8%	GFJY65E01AQ60L	292-36	7-262
		440	0	0	0	0%	GFJY65E01CJM64	1-231	35-262
cyclin B3	AB443443	802	270	0	270	34%	GE8SX9M02GFOJ4	271-368	704-802
cyclin E	AB378067	209	1276	617	659	611%	isotig01641	660-850	full

		209	1371	712	659	656%	isotig01640	660-850	full
		209	1446	787	659	692%	isotig01639	660-850	full
		209	3567	617	2896	1707%	isotig01638	2897-3087	full
		209	3608	712	2896	1726%	isotig01637	2897-3087	full
		209	3683	787	2896	1762%	isotig01636	2897-3087	full
DHHC-type zinc finger contiaining protein	AB378066	643	450	450	0	70%	isotig14108	353-1	29-381
discs overgrown	AB443442	287	3115	343	2772	1085%	isotig01394	344-630	full
		287	3211	439	2772	1119%	isotig01393	440-726	full
ecdysone receptor B1	AB536932	828	435	0	435	53%	isotig14153	129-364	593-828
EF1alpha	AB583234	2029	101	101	0	5%	contig12129	101-604	1-155, 239-421, 490-660
		2029	116	0	116	6%	contig12130	117-505	1466-1543, 1627-1794, 1884-2029
elongation factor	DQ630923	717	432	0	432	60%	contig09678	433-779	371-717
		717	910	910	0	127%	contig09671	911-1048	1-138
Ena/VASP	AB378069	200	561	199	362	281%	isotig15279	363-515	25-177
enhancer of zeste	AB378079	431	1938	1423	515	450%	isotig05120	1424-1579, 1637-1854	1-218, 276-431
		431	2076	1423	653	482%	isotig05119	1424-1579, 1637-1854	1-218, 276-431
expanded	AB378099	648	182	0	182	28%	GFJY65E01B9CAF	1-284	346-629
		648	0	0	0	0%	GFJY65E01DAZCK	full	63-343
fasciclin-like protein	DQ630929	768	1870	935	936	243%	isotig09432	936-1703	full
fmr	AB461422	1854	42	42	0	2%	isotig06512	130-1053	88-1011
		1854	0	0	0	0%	isotig17262	full	1284-1825
epidermal growth factor receptor	AB300616	3807	0	0	0	0%	isotig12088	full	2369-3450
		3807	275	0	275	7%	isotig18881	276-456	3625-3807
GB1-cadherin	AB190295	4945	38	38	0	1%	isotig04828	1164-4545	1-3382

		4945	125	0	125	3%	isotig10276	126-1688	3383-4945
GB2-cadherin	AB190296	4096	203	203	0	5%	GFCP6CO01ER8W2	1-176	1-176
		4096	0	0	0	0%	FQTBZRY01B5B7K	full	875-1011
grainy head	AB378081	826	1244	0	1244	151%	isotig10851	1-225	579-803
		826	0	0	0	0%	FQTBZRY02GC2DO	full	7-226
Gug gene corepressor Atro 3'	AB378078	192	581	101	480	303%	isotig14567	102-166, 232-288	6-70, 136-192
Gug gene corepressor Atro 5'	AB378077	179	1671	1011	660	934%	isotig09993	1012-1151	17-156
hedgehog	AB044709	2963	0	0	0	0%	GE8SX9M01BZRKW	full	2142-2471
hexokinase	DQ630934	432	1539	769	770	356%	isotig09401	770-1201	full
hippo	AB378070	632	993	136	857	157%	isotig03128	137-768	full
		632	1131	136	995	179%	isotig03127	137-768	full
		632	321	136	185	51%	isotig03129	137-640	1-504
hunchback	AB120735	2672	0	0	0	0%	GFJY65E01C2FLA	full	2062-2295
		2672	0	0	0	0%	GE8SX9M02GCICC	full	2323-2669
inhibitor of apoptosis protein	AB378071	542	1253	168	1085	231%	isotig03633	1086-1628	full
		542	2241	1156	1085	413%	isotig03632	1086-1527	102-543
Insulin receptor	AB557977	386	865	431	434	224%	isotig04919	435-783	full
		386	3991	3558	434	1034%	isotig04918	435-783	full
kibra	DC445461	677	464	464	0	69%	isotig12669	567-971	103-507
		677	0	0	0	0%	isotig19618	full	10-560
		677	0	0	0	0%	isotig13198	full	1-669
		677	256	0	256	38%	isotig19193	332-285	601-648
merlin	AB378073	539	3525	3174	351	654%	isotig07940	3175-3712	full
mob as tumor suppressor	AB378072	381	1482	424	1058	389%	isotig09892	1059-1439	full
Musashi	AB459508	354	345	345	0	97%	GFJY65E02G1KQY	346-415	1-70
nitric oxide synthase	AB477987	3535	0	0	0	0%	GE8SX9M02FRE69	full	2233-2676
		3535	0	0	0	0%	GFCP6CO01EGMNE	full	2299-2671
		3535	0	0	0	0%	GFJY65E01DHCQJ	full	1751-2105

		3535	163	0	163	5%	GFjY65E02G0F7L	1-254	3272-3525
Notch	AB635585	2304	0	0	0	0%	isotig14599	full	374-1132
		2304	0	0	0	0%	isotig12243	full	1595-2300
		2304	0	0	0	0%	GE8SX9M01BNVPA	full	1145-1566
		2304	194	194	0	8%	GFCP6CO01B89FU	198-229	4-35
orthodenticle1	AB468156	720	599	0	599	83%	isotig12009	1-519	200-707
period	AB375516	3552	0	0	0	0%	isotig11839	436-1044	101-701
		3552	0	0	0	0%	GFCP6CO01EOOUQ	18-150, 509-538	1999-2155
phosphatase and tensin	AB370293	490	870	460	410	178%	isotig11178	411-775	full
polycomb protein	AB444104	1333	503	0	503	38%	isotig14622	504-761	1062-1319
		1333	0	0	0	0%	GE8SX9M02FSJ8W	full	789-1059
Ras association family member	AB443439	442	3221	133	3088	729%	isotig05452	3089-3530	full
		442	133	133	0	30%	isotig05453	305-172	1-134
s6k	AB557979	497	2738	491	2247	551%	isotig08277	2248-2744	full
S9 ribosomal protein	DQ630939	552	218	82	136	39%	isotig06773	82-626	full
		552	408	82	326	74%	isotig03301	82-398	1-420
salvador	AB378074	347	170	170	0	49%	GFJY65E01DC652	171-477	16-322
semaphorin 2a	EF036538	1306	203	0	203	16%	GFJY65E01CQDHA	1-49	1256-1303
sex combs reduced	AB194276	1015	0	0	0	0%	FQTBZRY02F97XW	4-34, 112-261	382-531, 609-639
Target of rapamycin	AB557078	269	230	12	218	86%	GFCO6CO02JFLMZ	13-277	full
tgf alpha (EGFR ligand)	HM106520	520	1476	0	1476	284%	isotig10026	1-323	194-520
timeless	AB548625.1	5795	0	0	0	0	isotig11684	full	3597-4790
		5795	0	0	0	0	isotig12618	full	4793-5784
		5795	0	0	0	0	isotig13095	full	2685-3596
		5795	0	0	0	0	isotig10108	1-722, 737-954, 1040-1758	622-1339, 1357-1574, 1660-2378
		5795	0	0	0	0	isotig15714	full	1-591
		5795	0	0	0	0	GFJY65E01EDCUE	full	2382-2663

Ultrabithorax	AB194278	790	0	0	0	0%	GFJY65E02JHD6P	361-58, 34-1	197-495, 519-552
		790	0	0	0	0%	GE8SX9M01BU9CB	1-135, 157-356	362-496, 519-720
vasa	AB378065	1953	420	0	420	22%	isotig11874	1146-421	1228-1953
		1953	0	0	0	0%	isotig14543	full	439-1200
Warts kinase	AB300574	861	93	93	0	11%	isotig14894	641-1	19-659
wingless	AB044713	2298	0	0	0	0%	GE8SX9M01DIP5X	full	1610-2075
yorkie	AB378076	1021	403	403	0	39%	GFCP6CO03JM8ZY	404-507	1-102
