# Supplementary material for: Developmental Gene Discovery in a Hemimetabolous Insect: De Novo Assembly and Annotation of a Transcriptome for the Cricket Gryllus bimaculatus
Source: PLoS One. 2013 May 6;8(5):e61479. doi: 10.1371/journal.pone.0061479 (PMC3646015; doi:10.1371/journal.pone.0061479)
Supplement: Table S3 — FlyTF transcription factor orthologs identified in the G. bimaculatus transcriptome. BLAST (E-cutoff 1e-5) was used to search the G. bimaculatus transcriptome for orthologs to the transcription factors belonging to the FlyTF database [80]. (PDF) [file pone.0061479.s006.pdf]

Table S3

Transcription factors from the FlyTF database with putative orthologues identified in the *de novo G. bimaculatus* transcriptome.

AGO1	Camta	CG16778	CG4617	CG9305	Dcr-2	fd3F	jim
ab	cas	CG16779	CG4789	CG9416	Deaf1	Fen1	jing
abo	caz	CG16903	CG4882	CG9418	Dhc16F	fru	jumu
Acf1	cdc2	CG17209	CG5147	CG9425	Dip1	fry	kay
Ada2b	Cdk7	CG17829	CG5245	CG9437	dl	fs(1)h	Kdm4A
Alh	Cdk8	CG17912	CG5316	CG9705	DLP	ftz-f1	Kdm4B
alien	Cdk9	CG1832	CG5343	CG9817	dom	Gas41	ken
aop	cg	CG18619	CG5380	CG9890	Dp	GATAd	kin17
Arc42	CG10289	CG1965	CG5591	CG9932	Dp1	gce	king-tubby
ash1	CG10348	CG2712	CG5641	chinmo	DppIII	gcl	Kr-h1
ash2	CG10414	CG2790	CG5690	chm	Dref	gl	kto
Asx	CG10431	CG31211	CG5953	Chrac-14	Dsp1	gol	l(2)37Cg
Atac1	CG10565	CG31716	CG6129	Chrac-16	dys	grh	l(2)k10201
Atf6	CG10979	CG32121	CG6654	Chro	E(bx)	grn	l(2)NC136
aub	CG11414	CG32343	CG6686	cic	e(r)	gro	l(3)mbt
bab2	CG11456	CG3281	CG6701	Clk	e(y)1	grp	La
bap	CG11617	CG32830	CG6751	cnc	e(y)2	Gug	lack
Bap170	CG11710	CG32982	CG6765	Cog7	e(y)3	H	lds
Bap55	CG11876	CG3328	CG6769	CoRest	E(z)	h	lid
Bap60	CG12071	CG33695	CG6812	Cp190	E2f	hay	LIMK1
bbx	CG12162	CG33785	CG6905	crc	E2f2	Hcf	lin-52
bic	CG12236	CG33936	CG6907	CrebA	ear	hep	Lmpt
bigmax	CG12267	CG3407	CG7099	CrebB-17A	ecd	Hira	lola
Bin1	CG12299	CG34422	CG7339	CREG	EcR	HLH106	lolal
bip2	CG1233	CG3680	CG7368	crm	ECSIT	Hnf4	Mad
Bka	CG12370	CG3711	CG7556	croc	egg	HP1b	maf-S
bon	CG12769	CG3726	CG7785	crol	Eip74EF	hpo	mamo
br	CG13204	CG3735	CG7818	ct	Eip78C	Hr39	Mat1
brat	CG13458	CG3756	CG7839	CtBP	Eip93F	Hr4	Max
Brd8	CG13624	CG3815	CG7987	CTCF	EloA	Hr78	MBD-like
Brf	CG14200	CG3838	CG8152	CycC	Elongin-B	Hr96	MBD-R2
brk	CG14767	CG3909	CG8290	CycH	Elp3	Hsf	mbf1
brm	CG14962	CG40196	CG8359	CycT	emc	hth	Med
bs	CG15011	CG4042	CG8578	CYLD	ERR	Iswi	MED1
BtbVII	CG15270	CG4404	CG8765	D12	Ets97D	ix	MED11
bun	CG15436	CG4553	CG8909	d4	ewg	Jarid2	MED14
Caf1	CG1620	CG4557	CG8924	dalao	exd	JIL-1	MED15

MED16	Mtp	phtf	Rpb8	Spt6	tld
MED17	mTTF	piwi	Rpd3	Ssdp	tna
MED18	mtTFB1	Pms2	RpI1	Ssl1	Top2
MED20	mtTFB2	pnt	RpI12	Ssrp	Top3alpha
MED21	mus201	polybromo	RpI135	Stat92E	tou
MED22	mus308	Pop2	RpII140	stc	tral
MED23	mus309	ppl	RpII15	su(Hw)	Trax
MED24	Myb	pps	RpII18	su(s)	Trf2
MED25	N	Psc	RpII215	Su(var)2-10	Trl
MED27	Nap1	Psf2	RpII33	Su(var)205	Trn-SR
MED28	NC2alpha	psq	RpIII128	Su(var)3-9	trr
MED30	nej	pum	RpL40	Su(z)12	trsn
MED31	NELF-A	Pur-alpha	RpL7	sug	trx
MED4	NELF-B	put	sa	svp	ttk
MED6	Nelf-E	pygo	Sap30	Taf1	Tudor-SN
MED7	Nf-YA	pzg	Scamp	Taf10	Ubi-p63E
MED8	Nf-YB	r	Sce	Taf11	Unr
Meics	Nf-YC	Rab-RP4	Scm	Taf12	Usf
melt	Nipped-A	Rab1	Set2	Taf13	usp
MEP-1	Nipped-B	Rab10	Sfmbt	Taf2	Utx
Mes-4	nos	Rab11	sgg	Taf4	wash
Mes4	Not1	Rab2	sim	Taf5	wts
MESR4	Nufip	Rab26	sima	Taf6	Xbp1
Met	opa	Rab27	simj	Taf8	XNP
Mi-2	Orc1	Rab35	Sin3A	tai	Xpd
mib1	Orc2	Rab8	Sir2	tara	yki
Mio	Orc5	Rbf	Sirt2	Tbp	YL-1
mip120	osa	Rel	Sirt4	tefu	yps
mip130	ovo	rept	Sirt6	TFAM	zfh1
mip40	p53	Rfx	Sirt7	Tfb1	zfh2
Mitf	pad	Rga	skd	Tfb4	Zpr1
Mlh1	Parg	rhea	Smox	TfIIA-L	
mod(mdg4)	Parp	rig	Smr	TfIIB	
mor	Pbp49	rl	sno	TfIIEalpha	
MRG15	Pbp95	rn	Snr1	TfIIEbeta	
mrn	Pc	rno	Sp1	TfIIFalpha	
mRpL28	Pcf11	row	spel1	TfIIFbeta	
mRpL55	Pcl	RpA-70	spen	TfIIS	
Msh6	peb	Rpb10	spn-A	TH1	
msl-3	pfk	Rpb11	spn-E	Thd1	
MTA1-like	ph-d	Rpb4	Spt3	Tif-IA	
MTF-1	ph-p	Rpb5	spt4	tim	
mtg	pho	Rpb7	Spt5	tkv	
