# Supplementary material for: Developmental Gene Discovery in a Hemimetabolous Insect: De Novo Assembly and Annotation of a Transcriptome for the Cricket Gryllus bimaculatus
Source: PLoS One. 2013 May 6;8(5):e61479. doi: 10.1371/journal.pone.0061479 (PMC3646015; doi:10.1371/journal.pone.0061479)
Supplement: Table S4 — Selected signaling pathway genes identified in the G. bimaculatus transcriptome. Hit ID indicates if gene hits were found assembled reads (A) or singletons (S). Length (range) indicates the shortest and longest A or S hit sequences for each gene. Query organism was D. melanogaster for all cases. (PDF) [file pone.0061479.s007.pdf]

Table S4

Selected signaling pathway genes identified in the *de novo G. bimaculatus* transcriptome.

Process	# Hits	Hit ID (I/S)	Length (range)	Query Gene	Transcriptome Sequence Name(s)
HEDGEHOG					
CK1	1	A	3248	*Ck1 alpha*	isotig08262
	2	A	3402-3498	*dco*	isotig01394, isotig01393
	1	A	2691	*gish*	isotig08729
Cos2	1	A	4125	*cos*	isotig07930
Fused	1	A	1624	*fu*	isotig10451
TGFb	1	A	1625	*gbb*	isotig07565
GSK-3β	2	A, S	367-483	*sgg*	GFJY65E02I1Z50, isotig18361
Megalin	1	A	2667	*Cg42611*	isotig08756
Patched	2	S	328-411	*ptc*	GFJY65E02I1VDN, GFJY65E01ALZ8M
PKA	1	A	4812	*Pka-C1*	isotig07789
Smoothened	2	A	705	*smo*	isotig13374, isotig15392
Suppressor of fused	1	A	2625	*Su(fu)*	isotig08905
Slim b	1	A	4768	*slmb*	isotig04954
JAK/STAT					
AKT	1	A	2629	*Akt1*	isotig08797
Cb1	1	A	486	*Cb1*	isotig18303
CBP	4	A, S	200-1501	*nej*	isotig17362, isotig05855, GE8SX9M02I88X1, isotig13864
PIAS	2	A	4065-4260	*Su(var)2-10*	isotig04583, isotig04582
GRB	1	A	2371	*drk*	isotig00085
JAK	1	A	2719	*hop*	isotig04276

PI3K	1	A	5218	*Pi3K21B*	isotig07744
	1	A	1976	*Pi3K92E*	isotig08270
SHP2	1	S	266	*csw*	GE8SX9M02G96K3
SOCS	1	A	2289	*Socs16D*	isotig09205
	1	A	3530	*Socs44A*	isotig08127
	2	A	2127-2190	*Socs36E*	isotig05318, isotig05317
SOS	5	A, S	170-1931	*Sos*	isotig09775, GFJY65E01CUEPZ, GFCP6CO02F9P6M, GAP9EXG04D7UG1, isotig14668
Spred	2	A	1189-3475	*Spred*	isotig05180, isotig05181
STAM	2	S	315-520	*Stam*	GFJY65E02GH370, GE8SX9M02IFMFR
STAT	1	A	2243	*Stat92E*	isotig03185
NOTCH					
APH-1	1	A	4738	*aph-1*	isotig04141
CIR	1	A	1088	*CG6843*	contig11433
CtBP	3	A, S	239-624	*CtBP*	isotig16142, GE8SX9M01EF4BJ, FQTBZRY01BYCPR
Deltex	2	A	1825-2309	*dx*	isotig09973, isotig09188
disheveled	2	A	2448-5763	*dsh*	isotig07449, isotig07448
Groucho	3	A, S	211-515	*gro*	isotig17698, GFJY65E01DLKWU, GFJY65E02GG7B9
HATs	4	A, S	200-1501	*nej*	isotig17362, isotig05855, GE8SX9M02I88X1, isotig13864
HDAC	1	A	2212	*Rpd3*	isotig09325
Nicastrin	4	A	766-2581	*nct*	isotig03085, isotig03084, isotig05814, isotig05814
Notch	4	A, S	423-2816	*Notch*	isotig14599, GE8SX9M01BNVPA, isotig12243, isotig08601
Presenilin	2	A	1999-3017	*Psn*	isotig03035, isotig03036
PSE2	1	A	864	*pen-2*	isotig13452
SKIP	3	A, S	338-2107	*Bx42*	isotig05493, isotig05494, GFJY65E02IALF1
Tace	1	A	3117	*Tace*	isotig08377
WNT					
APC	4	S	208-470	*Apc*	GFCP6CO02IKY6E, GFCP6CO01CGKAB, GFJY65E01EDMSG, GFJY65E01D5QKT
Axin	2	A	1769-2651	*Axn*	isotig00276, isotig08771

beta-catenin	1	A	3974	*arm*	isotig05341
beta-TrCP	1	A	4768	*slmb*	isotig04954
CaMKII	2	A	1262-2572	*CaMKII*	isotig05571, isotig05572
CaN	1	A	3292	*CanB2*	isotig05734
CBP	4	A, S	200-1501	*nej*	isotig17362, isotig05855, GE8SX9M02I88X1, isotig13864
CK1	1	A	3248	*Ck1 alpha*	isotig08262
	2	A	3402-3498	*dco*	isotig01394, isotig01393
	1	A	2691	*gish*	isotig08729
CK2	2	A	3799-4012	*CkII beta*	isotig02546, isotig02545
CtBP	3	A, S	239-624	*CtBP*	isotig16142, GE8SX9M01EF4BJ, FQTBZRY01BYCPR
Cul1	4	A	3731-5498	*lin19*	isotig02458, isotig02457, isotig03607, isotig03606
Daam1	1	S	223	*DAAM*	FQTBZRY02HV44R
disheveled	2	A	2448-5763	*dsh*	isotig07449, isotig07448
Ebi1	1	A	2312	*ebi*	isotig09177
GSK-3β	2	A, S	367-483	*sgg*	GFJY65E02I1Z50, isotig18361
Groucho	3	A, S	211-515	*gro*	isotig17698, GFJY65E01DLKWU, GFJY65E02GG7B9
JNK	1	S	230	*bsk*	GFJY65E01CRM61
LRP5/6	2	S	259-493	*arr*	GFCP6CO01EVQLD, FQTBZRY02HHNYA
NLK	1	A	3303	*nmo*	isotig04244
PKA	1	A	4812	*Pka-C1*	isotig07789
PKC	1	A	4789	*Pkc53E*	isotig07795
PLC	1	S	329	*norpA*	GFJY65E01AO8M1
PP2A	3	A	1910-5172	*Pp2A-29B*	isotig02130, isotig02129, isotig09820
	1	A	1734	*mts*	isotig00164
Proc	1	A	1974	*por*	isotig09691
Protein52	1	A	1461	*pont*	contig15673
PS-1	2	A	1999-3017	*Psn*	isotig03035, isotig03036
Rac	1	A	2954	*Rac1*	isotig08497
Rbx1	2	S	459-480	*Roc1a*	GFCP6CO02GX3GB, GFCP6CO02I0JF4
RhoA	2	A	2482-3812	*Rho1*	isotig00258, isotig03933
rhomboid-7	1	A	3315	*rho-7*	isotig05079

ROCK2	8	A	853-4515	*rok*	isotig01612, isotig01613, isotig01614, isotig01615, isotig01616, isotig01617, isotig06106, isotig06107
Siah-1	2	A	1698-2386	*sina*	isotig09073, isotig10251
	8	A	3812-4068	*sinah*	isotig00589, isotig00588, isotig00587, isotig00586, isotig00585, isotig00584, isotig00583, isotig00582
SIP	1	A	587	*CG3226*	contig15490
Skp1	1	A	951	*skpF*	isotig12819
SMAD3	1	S	332	*Smox*	GFCP6CO01DS40Z
SMAD4	1	A	729	*Med*	isotig15042
Stbm	1	A	2916	*Vang*	isotig08532
Wif-1	2	A	1568-1581	*shf*	isotig02624, isotig02623
TGF-BETA					
ActivinRI	1	A	2267	*babo*	isotig09236
Cul1	4	A	3731-5498	*lin19*	isotig02458, isotig02457, isotig03607, isotig03606
DP1	1	A	3452	*tfdp1a*	isotig08163
E2F4/5	4	A	1708-1929	*e2f4*	isotig00805, isotig00806, isotig00807, isotig00808
ERK	1	A	799	*rl*	isotig14164
Id	1	S	201	*emc*	FQTBZRY02G5SHM
TGFb	1	A	1625	*gbb*	isotig07565
p107	2	A	6434-6542	*Rbf*	isotig04489, isotig04488
p300	4	A, S	200-1501	*nej*	isotig17362, isotig05855, GE8SX9M02I88X1, isotig13864
p70S6K	1	A	3234	*S6K*	isotig08277
PP2A	3	A	1910-5172	*Pp2A-29B*	isotig02130, isotig02129, isotig09820
	1	A	1734	*mts*	isotig00164
Rbx1	2	S	459-480	*Roc1a*	GFCP6CO02GX3GB, GFCP6CO02I0JF4
RhoA	2	A	2482-3812	*Rho1*	isotig00258, isotig03933
ROCK1	8	A	853-4515	*rok*	isotig01612, isotig01613, isotig01614, isotig01615, isotig01616, isotig01617, isotig06106, isotig06107
SARA	1	A	2592	*Sara*	isotig08835
Skp1	1	A	951	*skpF*	isotig12819
Smad1/5/8	1	A	2120	*Mad*	isotig09444
Smad2/3	1	S	332	*Smox*	GFCP6CO01DS40Z

Smad4	1	A	729	*Med*	isotig15042
Smurf1/2	1	A	4308	*lack*	isotig07879
MAPK					
Boss	1	A	3134	*boss*	isotig08354
Csw	1	S	266	*csw*	GE8SX9M02G96K3
Drk	1	A	2371	*drk*	isotig00085
Dsor1	1	A	3545	*Dsor1*	isotig08121
Egfr	1	A	1099	*Egfr*	isotig12088
Gap1	2	S	280-358	*Gap1*	GE8SX9M02HTUD8, GFJY65E01B2IBY
Phl	1	A	4282	*phl*	isotig07892
Pointed	1	S	314	*pnt*	GFCP6CO01CJJKD
Ras85D	2	A	2078-2467	*Ras85D*	isotig09494, isotig08979
Rolled	1	A	799	*rl*	isotig14164
Sos	5	A, S	170-1931	*Sos*	isotig09775, GFJY65E01CUEPZ, GFCP6CO02F9P6M, GAP9EXG04D7UG1, isotig14668
Ts1	1	S	174	*ts1*	GFCP6CO02G92YK
Yan	1	A	4007	*aop*	isotig07960
HIPPO					
cyclinE	6	A	1521-3799	*CycE*	isotig01638, isotig01637, isotig01636, isotig01641, isotig01640, isotig01639
Dco	2	A	3402-3498	*dco*	isotig01394, isotig01393
diap1	2	A	1796-2785	*th*	isotig03633, isotig03632
Expanded	2	S	306-466	*ex*	GFJY65E01B9CAF, GFJY65E01DAZCK
Fat	1	A	716	*ft*	isotig15250
Hippo	3	A	953-1763	*hpo*	isotig03127, isotig03128, isotig03129
homothorax	5	S	153-234	*hth*	FQTBZRY01D5WGD, FQTBZRY01AYECO, FQTBZRY02GY7HW, FQTBZRY02JLK81, FQTBZRY02FHQV8
Kibra	4	A	365-974	*kibra*	isotig12669, isotig19618, isotig19193, isotig13198
Merlin	1	A	1313	*Mer*	isotig11307
Mob as tumor suppressor	1	A	1862	*mats*	isotig09892
Salvador	1	S	479	*sav*	GFJY65E01DC652

Warts	1	S	300	*wts*	GFJY65E01AT7SH
yorkie	1	S	507	*yki*	GFCP6CO02JM8ZY
