# Supplementary material for: Developmental Gene Discovery in a Hemimetabolous Insect: De Novo Assembly and Annotation of a Transcriptome for the Cricket Gryllus bimaculatus
Source: PLoS One. 2013 May 6;8(5):e61479. doi: 10.1371/journal.pone.0061479 (PMC3646015; doi:10.1371/journal.pone.0061479)
Supplement: Table S5 — Selected gametogenesis genes identified in the G. bimaculatus transcriptome. Hit ID indicates if gene hits found were assembled reads (A) or singletons (S). Length (range) indicates the shortest and longest A or S hit sequences for each gene. Groups of hits of a given color indicate transcriptome sequences that mapped to the same overlapping region of the BLAST target (putative SNPs or isoforms); hits of different colors indicate transcriptome sequences that map to different, non-overlapping regions of the BLAST target. Query organism was D. melanogaster for all cases. (PDF) [file pone.0061479.s008.pdf]

Table S5

Selected gametogenesis genes identified in the *de novo G. bimaculatus* transcriptome

Process	# Hits	Hit ID (A/S)	Length (range)	Query Gene	Transcriptome Sequence Names
**SPERMATOGENESIS**[1]					
TRANSCRIPTION FACTORS					
Enchancer of bithorax	1	A	4242	*E(bx)*	contig15318
eyes absent	1	S	401	*eya*	GFJY65E01EO7KL
Heat shock factor	4	A	3119-3268	*Hsf*	isotig01705, isotig01704, isotig01703, isotig01702
maleless	1	A	3818	*mle*	isotig05146
MBD-like	7	A	694-1211	*MBD-like*	isotig01061, isotig01060, isotig01064, isotig01063, isotig01062, isotig01066, isotig01065
Myb oncogene-like	1	A	3771	*Myb*	isotig08042
Rfx	1	A	1001	*Rfx*	isotig12547
TATA box binding protein-related factor 2	3	A, S	399-3469	*Trf2*	GFCP6CO01B8937, isotig01886, isotig01885
CYTOSKELETON					
Adenomatous popylosis coli tumor suppressor homolog (APC)	4	S	208-470	*Apc*	GFCP6CO02IKY6E, GFCP6CO01CGKAB, GFJY65E01EDMSG, GFJY65E01D5QKT
Adenomatous popylosis coli tumor suppressor homolog 2	2	S	315-470	*Apc2*	GFCP6CO02IKY6E, GFJY65E01D5QKT
beta tubulin	2	A	546-950	Btub56D	contig00262, contig00455

[1] Although we did not include cDNA derived from adult testes in our sequencing libraries, we nonetheless chose to perform manual annotation of genes known to be involved in *D. melanogaster* spermatogenesis since the creation of the testis germ line stem cell niche takes place during embryogenesis in *D. melanogaster* (Aboïm AN (1945) Développement embryonnainre et post-embryonnaire des gonades normales et agamétiques de *Drosophila melanogaster*. Revue Suisse de Zoologie 3: 53-154; Le Bras S, Van Doren M (2006) Development of the male germline stem cell niche in Drosophila. Developmental BIology 294: 92-103.) and in orthopterans 3. Nelsen OE (1931) Life cycle, sex differentiation, and testis development in *Melanoplus differentialis* (Acrididae, Orthoptera). Journal of Morphology 51: 467-525.)

cortactin	1	A	1147	*Cortactin*	isotig11852
diaphanous	1	S	237	*dia*	FQTBZRY01CIL7E
jaguar	3	A	958-2609	*jar*	isotig12791, isotig12012, isotig08822
Kinesin like protein at 61F	3	A	2102-3639	*Klp61F*	isotig01563, isotig01564, isotig01565
Myosin 31DF	2	A	1018-1306	*Myo31DF*	isotig11312, isotig12459
peanut	1	A	1957	*pnut*	isotig09723
Rac1	1	A	2954	*Rac1*	isotig08497
Spectrin 1	4	A	409-2155	*α-Spec*	isotig09397, isotig10052, isotig15468, isotig19330
spindle assembly abnormal 6	1	A	2655	*sas-6*	isotig05533
subito	1	A	2615	*sub*	contig14686
twinstar	2	A	513-2077	*tsr*	isotig00493, isotig00494
zipper	2	A	3077-3958	*zip*	isotig05158, isotig08407
OTHER PROCESSES IN SPERMATOGENESIS					
armitage	1	A	4095	*armi*	isotig07934
asterless	1	A	3788	*asl*	isotig08040
aubergine	2	A	2674-2784	*aub*	isotig07461, isotig07462
boule	1	S	203	*bol*	GFJY65E01B4FFK
bride of sevenless	1	A	3134	*boss*	isotig08354
Btk family kinase at 29A	2	A	915-1545	*Btk29A*	isotig06869, isotig10647
Bub1-related kinase	1	A	4209	*BubR1*	isotig07912
Calmodulin	3	A	1591-1698	*Cam*	isotig00266,isotig00265, isotig00264
capsuleen	2	A	3725-3816	*csul*	isotig01229, isotig01228
cdc2	1	A	2078	*cdc2*	isotig03292
courtless	1	A	1123	*crl*	isotig11993
Cyclin A	1	A	3049	*CycA*	isotig03226
Cytochrome c proximal	1	A	636	*Cyt-c-p*	contig10573
Cytochrome c distal	1	A	778	*Cyt-c-d*	isotig14404
Dynamin related protein 1	4	A	908-3502	*Drp1*	isotig13131, isotig01328, isotig01327, isotig01326
effete	2	A	3342-4080	*eff*	isotig01782, isotig01780
Fmr1	2	A	1038-1053	*Fmr1*	isotig06512, isotig06513
Fps oncogene analog	1	A	328	*Fps85D*	isotig19747
glass bottom boat	1	A	1625	*gbb*	isotig07565

gilgamesh	1	A	2691	*gish*	isotig08729
hephaestus	2	S	201-359	*heph*	GE8SX9M01A0TGF, FQTBZRY02F9D2F
Ice	2	A	1620-1800	*Ice*	isotig04366, isotig10455
karyopherin α1	1	A	1309	*Kap-α1*	isotig11303
loquacious	1	A	2867	*loqs*	isotig02873
Microcephalin	2	A	3384-4822	*MCPH1*	isotig04588, isotig04589
Myt1	1	A	3433	*Myt1*	isotig04225
Nedd2-like caspase	1	A	2900	*Nc*	isotig03487
parkin	2	A	3339-3502	*park*	isotig04723, isotig04722
pavarotti	2	A	2221-2661	*pav*	isotig03050, isotig03049
pelota	1	A	922	*pelo*	contig17247
piwi	1	A	1277	*piwi*	isotig11428
pole hole	1	A	4282	*phl*	isotig07892
punt	1	S	441	*put*	GE8SX9M01B9MGK
Rab-protein 11	1	A	2448	*Rab11*	isotig00835
Rheb	1	A	953	*Rheb*	contig21414
shotgun	1	A	4583	*shg*	isotig04828
shut down	2	A	2449-3029	*shu*	isotig04931, isotig04930
string	1	A	911	*stg*	isotig13103
Syntaxin 5	3	A	2683-3493	*Syx5*	isotig01824, isotig01823, isotig01825
transformer 2	1	A	836	*tra2*	contig12123
terribly reduced optic lobes	1	A	690	*trol*	isotig15574
uncoordinated	1	A	2116	*unc*	isotig09457
vav	1	A	2068	*vav*	isotig09529
ypsilon schachtel	1	A	2601	*yps*	isotig03079
**OOGENESIS**					
MAINTENANCE AND DIVISION OF GERM LINE STEM CELLS					
armadillo	1	A	3974	*arm*	isotig05341
Axin	2	A	1769-2651	*Axn*	isotig00276, isotig08771
Dicer-1	1	A	2177	*Dcr-1*	isotig09376
dishevelled	2	A	2448-5763	*dsh*	isotig07449, isotig07448
effete	2	A	3342-4080	*eff*	isotig01782, isotig01780

fused	1	A	1624	*fu*	isotig10451
karst	16	A, S	140-568	*kst*	GFJY65E01C8HCB, GE8SX9M01D9LON, GFJY65E01EPKW2, GE8SX9M01D913W, FQTBZRY01EKMIL, GE8SX9M01AEJPJ, GFCP6CO01BN88A, GE8SX9M01ASUJ7, GFJY65E02HJ33N, isotig18880, GFCP6CO02F8AKG, GFCP6CO02GAOJB, isotig07261, GFCP6CO01AQ9N2, FQTBZRY02J3ED4, FQTBZRY01DAFOD
loquacious	1	A	2867	*loqs*	isotig02873
ovarian tumor	2	A	2393-2483	*out*	isotig05114, isotig05113
pelota	1	A	922	*pelo*	contig17247
piwi	1	A	1277	*piwi*	isotig11428
pumilio	3	A, S	412-624	*pum*	isotig04477, isotig04476, GFJY65E02G1R75
sans fille	1	A	1511	*snf*	isotig10698
shaggy	1	A	483	*sgg*	isotig18361
shavenbaby	1	A	795	*ovo*	isotig14222
shut down	2	A	2449-3029	*shu*	isotig04931, isotig04930
vasa	2	A	765-1146	*vas*	isotig14543, isotig11874
OOCYTE DETERMINATION AND FORMATION OF AP AXIS					
4EHP	1	A	1414	*4EHP*	isotig01556
alpha Spectrin	4	A	409-2155	*α-Spec*	isotig09397, isotig10052, isotig15468, isotig19330
beta-Tubulin at 56D	2	A	546-950	*Btub56D*	contig00262, contig00455
Bicaudal C	2	A	854-1435	*BicC*	isotig06390, isotig06389
Bicaudal D	2	A	687-1014	*BicD*	isotig12488, isotig15621
cAMP-dependent protein kinase 1	1	A	4812	*Pka-C1*	isotig07789
COP9 complex homolog subunit 5	2	A	1032-1284	*CSN5*	contig13654, isotig11391
cornichon	1	A	1733	*cni*	isotig05694
Dynein heavy chain 64C	8	A, S	344-1706	*Dhc64C*	isotig15021, isotig12385, isotig18811, GFJY65E02JTGDA, GFJY65E01CXFIZ, isotig13703, isotig10229, isotig10644
Dystroglycan	2	S	293-342	*Dg*	GFCP6CO01C30LP, GFCP6CO01BPUA2
egalitarian	2	A	878-1634	*egl*	isotig13386, isotig10415
egghead	1	A	796	*egh*	isotig14205
exuperantia	2	A	3152-3225	*exu*	isotig04764, isotig04765

Helicase at 25E	2	S	277-341	*Hel25E*	GFJY65E01EGNY3, GE8SX9M01BJ16P
hu-li tai shao	6	A	2255-2885	*hts*	isotig01647, isotig01646, isotig01645, isotig01644, isotig01643, isotig01642
Kinesin heavy chain	2	A	3918-7009	*Khc*	isotig04492, isotig04493
licorne	1	A	2845	*lic*	contig18303
lkb1	2	A	3048-3216	*lkb1*	isotig01200, isotig01199
maelstrom	1	A	2668	*mael*	isotig06013
okra	1	A	1794	*okr*	isotig10034
par-1	1	A	889	*par-1*	isotig07610
par-6	1	A	3994	*par-6*	isotig07961
pipsqueak	1	A	1991	*Rab-6*	isotig09661
tudor	1	A	3025	*spn-E*	contig00220
FORMATION OF DV AXIS					
cappuccino	2	A	817-866	*capu*	isotig06798, isotig06799
orb	1	A	4765	*orb*	isotig00462
pipe	1	A	6608	*pip*	isotig07697
squid	1	A	1546	*sqd*	isotig00544
trailer hitch	2	A	263-493	*tral*	isotig18126, isotig07398
ACTING EARLY IN FOLLICLE CELLS (DORSAL GROUP)					
big brain	3	S	298-515	*bib*	GE8SX9M01BXNN0, GFJY65E01CFBEX, GFCP6CO01EV5QZ
bunched	1	A	869	*bun*	isotig13467
Chorion factor 2	1	S	147	*Cf2*	GFCP6CO01DSOAR
corkscrew	1	S	266	*csw*	GE8SX9M02G96K3
dodo	2	A	1975-1994	*dod*	isotig05499, isotig05500
broad	1	A	904	*br*	isotig13160
torpedo	1	A	1099	*Egfr*	isotig12088
Ets at 97D	1	A	2149	*Ets97D*	isotig05797
kibra ortholog	1	A	974	*kibra*	isotig12669
mago nashi	1	A	1021	*mago*	isotig12375
Notch	4	A, S	423-2816	*Notch*	isotig14599, GE8SX9M01BNVPA, isotig12243, isotig08601
pointed	1	S	314	*pnt*	GFCP6CO01CJJKD
Rac1	1	A	2954	*Rac1*	isotig08497

Ras oncogene at 85D	2	A	2078-2467	*Ras85D*	isotig09494, isotig08979
rolled	1	A	799	*rl*	isotig14164
singed	1	S	239	*sn*	GE8SX9M01EZ3K3
TERMINAL GENES					
SHC-adaptor protein	2	A	2374-2640	*Shc*	isotig05081, isotig05082
torso-like	1	S	174	*ts1*	GFCP6CO02G92YK
LIGANDS, RECEPTORS & EFFECTORS					
hopscotch	1	A	2719	*hop*	isotig04276
Keren	1	A	1803	*Krn*	isotig10026
kugelei	1	A	729	*kug*	isotig15037
Medea	1	A	729	*Med*	isotig15042
Mothers against dpp	1	A	2120	*Mad*	isotig09444
Protein tyrosine phosphastase 69D	2	A, S	471-1475	*Ptp69D*	isotig10837, GFCP6CO02HK6UL
punt	1	S	441	*put*	GE8SX9M01B9MGK
saxophone	1	A	4561	*sax*	isotig07822
shotgun	1	A	4583	*shg*	isotig04828
Star	1	A	4011	*S*	isotig07955
STAT	1	A	2243	*Stat92E*	isotig03185
GENES AFFECTING CYTOSKELETON					
adnormal spindle	1	A	6563	*asp*	isotig07699
alpha actinin	1	A	2837	*Actn*	isotig08592
Btk family kinase at 29A	2	A	915-1545	*Btk29A*	isotig06869, isotig10647
capulet	1	A	3379	*capt*	isotig04236
Cdc42	1	A	2958	*Cdc42*	isotig03915
Ced-12	1	A	3012	*Ced-12*	isotig08450
chromosome bows	1	A	1067	*chb*	isotig12228
sticky	1	A	3121	*sti*	isotig08364
Cortactin	1	A	1147	*Cortactin*	isotig11852
diaphanous	2	S	237-429	*dia*	FQTBZRY01CIL7E, GFJY65E01CBNCA
genghis khan	1	A	2408	*gek*	isotig09046
jaguar	3	A	958-2609	*jar*	isotig12791, isotig12012, isotig08822
kette	1	A	5316	*Hem*	isotig07736
Kinesin associated protein 3	1	A	3027	*Kap3*	contig12721
klarsicht	1	A	1805	*klar*	isotig10023

Lamin	1	A	1757	*Lam*	contig17155
Lissencephaly	1	A	4309	*Lis-1*	isotig02186
mushrom body defect	1	A	2026	*mud*	contig12641
rho-type guanine exchange factor	8	A, S	234-1847	*rtGEF*	GFJY65E01DWNJO,GFJY65E01DDZGX, isotig00295, isotig00293, GE8SX9M02JKH1C, GE8SX9M01EGTZF, GFCP6CO01CRFSJ, GE8SX9M02F0G9A
short stop	3	A	673-1571	*shot*	isotig13049, isotig10577, isotig15743
spaghetti squash	3	A	616-1053	*sqh*	contig15080, isotig00107, isotig00106
Src oncogene at 42A	1	A	1787	*Src42A*	isotig04219
subito	1	A	2615	*sub*	contig14686
Suppressor of profilin 2	1	A	1695	*Sop2*	isotig06657
twinstar	2	A	513-2077	*tsr*	isotig00493, isotig00494
washout	1	A	608	*wash*	isotig07224
zipper	1	A	3958	*zip*	isotig05158
OTHER GENES INVOLVED IN OOGENESIS					
altered disjunction	3	A	3259-3423	*ald*	isotig03616, isotig03615, isotig03614
archipelago	1	A	4393	*ago*	isotig00333
chiffon	1	A	3144	*chif*	isotig08349
Cyclin-dependent kinase 7	1	A	2272	*Cdk7*	isotig02269
Cyclin-dependent kinase subunit30A	1	A	1011	*Cks30A*	isotig06131
Cyclin E	6	A	1521-3799	*CycE*	isotig01638, isotig01637, isotig01636, isotig01641, isotig01640, isotig01639
double parked	1	A	5242	*dup*	isotig07741
E2F transcription factor	1	A	918	*E2f*	isotig13069
geminin	1	A	921	*geminin*	isotig04440
imaginal discs arrested	1	A	5406	*ida*	isotig07735
loki	1	A	2488	*lok*	isotig05744
meiotic 41	1	A	1228	*mei-41*	isotig11599
Microcephalin	2	A	3384-4822	*MCPH1*	isotig04588, isotig04589
morula	2	A	1648-1877	*mr*	isotig09875, isotig10364
mutagen-sensitive 209	1	A	1396	*mus209*	isotig00238
Myb oncogene-like	1	A	3771	*Myb*	isotig08042
Myt1	1	A	3433	*Myt1*	isotig04225
pitchoune	1	A	3126	*pit*	isotig04252
sarah	1	A	3505	*sra*	isotig08135
twins	2	A	2511-3747	*tws*	isotig04782, isotig04783
abstrakt	1	A	1429	*abs*	isotig10965

anterior open	1	A	4007	*aop*	isotig07960
aubergine	2	A	2674-2784	*aub*	isotig07461, isotig07462
Autophagy-specific gene 1	1	A	1467	*Atg1*	contig16688
basket	1	S	230	*bsk*	GFJY65E01CRM61
blistered	2	S	233-241	*bs*	GE8SX9M02GMAG7, GFJY65E02FKS1J
brainiac	1	A	1870	*brn*	isotig09883
Bruce	1	A	4923	*Bruce*	isotig07779
capsuleen	2	A	3725-3816	*csul*	isotig01229, isotig01228
Calmodulin-binding protein related to a Rab3 GDP/GTP exchange protein	2	A	1233-2207	*Crag*	isotig07677, isotig09331
combgap	1	A	3604	*cg*	isotig08105
Cyclic-AMP response element binding protein A	1	A	3290	*CrebA*	isotig08237
C-terminal binding protein	3	A, S	239-624	*CtBP*	isotig16142, GE8SX9M01EF4BJ, FQTBZRY01BYCPR
cut	1	S	247	*ct*	FQTBZRY02GRN27
Death related ced-3/Nedd2-like protein	1	A	2732	*Dredd*	isotig08688
Ecdysone-induced protein 63E	2	A	3994-4024	*Eip63E*	isotig02121, isotig02120
ecdysoneless	4	A, S	372-3069	*ecd*	isotig17485, isotig19531, GFCP6CO01D3B2B, isotig08412
eggless	2	A	2986-3019	*egg*	isotig04831, isotig04830
extra macrochaetae	1	S	201	*emc*	FQTBZRY02G5SHM
fat facets	5	A	1816-3259	*faf*	isotig01188, isotig01187, isotig01186, isotig01185, isotig01184
fruitless	2	A	1313-1618	*fru*	isotig06010, isotig06009
G protein-coupled receptor kinase 2	1	A	1632	*Gprk2*	isotig00416
G protein oα 47A	1	A	2901	*G-oα47A*	isotig05513
poly U binding factor 68kD	2	A	3724-3736	*pUf68*	isotig01566, isotig01567
Heat shock factor	4	A	3119-3268	*Hsf*	isotig01705, isotig01704, isotig01703, isotig01702
Heat-shock-protein-70	3	A	2209-2595	*Hsp70*	isotig09115, isotig00207, isotig00208
Hepatocyte growth factor regulated tyrosine kinase substrate	4	A, S	344-583	*Hrs*	isotig16755, GE8SX9M02I536Z, GE8SX9M01EC494, GE8SX9M01BQDGK
hephaestus	2	S	201-359	*heph*	GE8SX9M01A0TGF, FQTBZRY02F9D2F
Ice	2	A	1620-1800	*Ice*	isotig04366, isotig10455
jing	2	S	366-427	*jing*	GFCP6CO01CJPNC, GE8SX9M02FPBO4
jumeau	1	A	3251	*jumu*	isotig08268
leonardo	2	A	3053-3220	*14-3-3ζ*	isotig03712, isotig03711
lethal (2) giant larvae	2	A	1879-2573	*l(2)gl*	contig15364, contig15365
Lipid storage droplet-2	1	A	1861	*Lsd-2*	isotig06100
Liprin-α	3	A	982-1158	*Liprin-α*	isotig03903, isotig03902, isotig03901

maternal expression at 31B	1	A	3408	*me31B*	isotig00511
Merlin	1	A	4062	*Mer*	isotig07940
Methoprene-tolerant	1	S	420	*Met*	GFCP6CO01DP0NH
microtubule star	1	A	1734	*mts*	isotig00164
mini spindles	2	A	2315-4784	*msps*	isotig07797, isotig09181
misshapen	1	S	327	*msn*	GE8SX9M01DYBJ8
moira	1	A	2758	*mor*	isotig08664
Nedd2-like caspase	1	A	2900	*Nc*	isotig03487
nicastrin	2	A	2050-1886	*nct*	isotig03085, isotig03084
Niemann-Pick type C-2a	1	A	1095	*Npc2a*	contig15402
Nucleolar protein at 60B	1	A	878	*Nop60 B*	contig09572
O-fucosyltransferase 1	1	A	3328	*O-fut1*	isotig08223
Ornithine decarboxylase antizyme	1	A	2621	*Oda*	isotig08802
PDGF- and VEGF-receptor related	1	A	2602	*Pvr*	isotig08816
pollux	1	A	3676	*plx*	isotig08081
polyhomeotic distal	1	A	1608	*ph-d*	isotig10480
polyhomeotic proximal	1	S	514	*ph-p*	GFCP6CO02H0634
Presenilin	2	A	1999-3017	*Psn*	isotig03035, isotig03036
Rab-protein 5	2	A	3300-3532	*Rab5*	isotig02948, isotig02947
Rab-protein 11	1	A	2448	*Rab11*	isotig00835
rotund	1	S	186	*rn*	FQTBZRY02G28N8
scribbled	2	A, S	427-696	*scrib*	isotig15514, GE8SX9M01C2HSN
skittles	1	S	311	*sktl*	GE8SX9M01DH70U
SNF1A/AMP-activated protein kinase	1	A	2566	*SNF1A*	isotig05865
Snf5-related 1	1	A	3127	*Snr1*	isotig08358
spinster	1	A	3143	*spin*	isotig00443
SH2 ankyrin repeat kinase	1	A	3892	*shark*	isotig07997
strawberry notch	2	A	2330-2459	*sno*	isotig09159, isotig08990
suppressor of Hairy wing	2	A	1790-1907	*su(Hw)*	isotig01368, isotig01367
Suppressor of variegation 3-3	2	A, S	335-422	*Su(var)3-3*	isotig19729, GFCP6CO01BGKBV
Syntaxin 1A	1	A	4416	*Syx1A*	isotig04870
TATA box binding protein-related factor 2	2	A	3377-3469	*Trf2*	isotig01886, isotig01885
TBP-associated factor 1	1	A	5541	*Taf1*	isotig04746
terribly reduced optic lobes	1	A	690	*trol*	isotig15574
Trithorax-like	1	A	1267	*Trl*	isotig11437
warts	1	A	734	*wts*	isotig14894
widerborst	1	A	2141	*wdb*	contig21405

1. Aboïm AN (1945) Développement embryonnainre et post-embryonnaire des gonades normales et agamétiques de *Drosophila melanogaster*. Revue Suisse de Zoologie 3: 53-154.
2. Le Bras S, Van Doren M (2006) Development of the male germline stem cell niche in Drosophila. Developmental BIology 294: 92-103.
3. Nelsen OE (1931) Life cycle, sex differentiation, and testis development in Melanoplus differentialis (Acrididae, Orthoptera). Journal of Morphology 51: 467-525.
