# Supplementary material for: Developmental Gene Discovery in a Hemimetabolous Insect: De Novo Assembly and Annotation of a Transcriptome for the Cricket Gryllus bimaculatus
Source: PLoS One. 2013 May 6;8(5):e61479. doi: 10.1371/journal.pone.0061479 (PMC3646015; doi:10.1371/journal.pone.0061479)
Supplement: Table S6 — Selected developmental process genes identified in the G. bimaculatus de novo transcriptome assembly. Hit ID indicates if gene hits found were assembled reads (A) or singletons (S). Length (range) indicates the shortest and longest A or S hit sequences for each gene. Groups of hits of a given color indicate transcriptome sequences that mapped to the same overlapping region of the BLAST target (putative SNPs or isoforms); hits of different colors indicate transcriptome sequences that map to different, non-overlapping regions of the BLAST target. Query organism was D. melanogaster for all cases. (PDF) [file pone.0061479.s009.pdf]

Table S6

Selected developmental process genes identified in the *de novo G. bimaculatus* transcriptome.

Process	# Hits	Hit ID (A/S)	Length (range)	Query Gene	Transcriptome Sequence Name(s)
**MATERNAL GENES**					
ANTERIOR GROUP					
bicoid interacting protein 1	1	A	1040	*Bin1*	isotig03457
exuperantia	2	A	3152-3225	*exu*	isotig04765, isotig04764
staufen	3	A	1287-1442	*stau*	isotig03172, isotig03173, isotig03174
POSTERIOR GROUP					
armitage	1	A	4095	*armi*	isotig07934
Bruno	1	A	1676	*aret*	isotig10307
cappuccino	2	A	817-866	*capu*	isotig06798, isotig06799
fat facets	5	A	1816-3259	*faf*	isotig01188, isotig01187, isotig01186, isotig01185, isotig01184
Moesin	1	A	4272	*Moe*	isotig00886
mago nashi	1	A	1021	*mago*	isotig12375
par-1	1	A	889	*par-1*	isotig07610
pipsqueak	2	A, S	337-430	*psq*	isotig19171, GFCP6CO01CETJB
pumilio	3	A, S	412-624	*pum*	isotig04477, isotig04476, GFJY65E02G1R75
orb	1	A	4765	*orb*	isotig00462
Rabenosyn-5	1	A	1853	*Rbsn-5*	isotig09916
staufen	3	A	1287-1442	*stau*	isotig03172, isotig03173, isotig03174
tudor	2	A	4146-5784	*tud*	isotig07719, isotig07925
vasa	2	A	765-1146	*vas*	isotig14543, isotig11874
ypsilon schachtel	1	A	2601	*yps*	isotig03079
TERMINAL GROUP					
capicua	2	S	314-438	*cic*	GE8SX9M02IXJOG, GE8SX9M01D8UIJ

corkscrew	1	S	266	*csw*	GE8SX9M02G96K3
pole hole	1	A	4282	*phl*	isotig07892
Ras oncogene at 85D	2	A	2078-2467	*Ras85D*	isotig09494, isotig08979
rolled	1	A	799	*rl*	isotig14164
torso-like	1	S	174	*ts1*	GFCP6CO02G92YK
DORSAL GROUP					
cactus	4	A	3168-4301	*cact*	isotig02364, isotig02362, isotig02363, isotig02361
cappuccino	2	A	817-866	*capu*	isotig06798, isotig06799
cornichon	1	A	1733	*cni*	isotig05694
capicua	2	S	314-438	*cic*	GE8SX9M02IXJOG, GE8SX9M01D8UIJ
dorsal	5	A, S	325-810	*dl*	isotig14031, GE8SX9M02HRGAV, GFJY65E02GK63W, GFJY65E02FIMPE, GE8SX9M01CGCYQ
Egfr	1	A	1099	*Egfr*	isotig12088
gastrulation-defective	1	A	862	*gd*	isotig13529
Myd88	1	A	2079	*Myd88*	isotig09497
orb	1	A	4765	*orb*	isotig00462
pelle	2	A	3507-4221	*pll*	isotig02382, isotig02381
pipe	1	A	6608	*pip*	isotig07697
spatzle	1	A	2006	*spz*	isotig09642
squid	1	A	1546	*sqd*	isotig00544
Toll	1	A	2125	*Tl*	isotig09438
zucchini	1	A	1455	*zuc*	isotig00915
**ZYGOTICALLY TRANSCRIBED GENES**					
cap-n-collar	2	A	1549-2281	*cnc*	isotig05578, isotig05577
crocodile	2	A	890-966	*croc*	isotig06650, isotig06649
Tenascin major	7	A, S	200-833	*Ten-m*	GFJY65E01CUG9F, GE8SX9M01AOG18, GFCP6CO01DGZ87, FQTBZRY01EVWST, GFCP6CO02HATIX, GFCP6CO02G16S1, isotig13797
C-terminal binding protein	3	A, S	239-624	*CtBP*	isotig16142, GE8SX9M01EF4BJ, FQTBZRY01BYCPR
domeless	1	A	927	*dome*	isotig12992
eyelid	1	A	2298	*osa*	isotig09196
ftz transcription factor 1	1	S	397	*ftz-f1*	GFCP6CO02HU50W
hopscotch	1	A	2719	*hop*	isotig04276

marelle	1	A	2243	*Stat92E*	isotig03185
Rpd3	1	A	2212	*Rpd3*	isotig09325
shuttle craft	1	A	4369	*stc*	isotig07864
Sir2	1	A	2334	*Sir2*	contig14671
squid	1	A	1546	*sqd*	isotig00544
